# Potential applications of artificial intelligence in pain management: a scoping review

**DOI:** 10.3389/fpain.2026.1871943

**Published:** 2026-08-26

**Authors:** Dmitriy Viderman, Sultan Kalikanov, Meiram Tungushpayev, Min-Ho Lee, Dong-Ok Won

**Affiliations:** 1 Department of Surgery, School of Medicine, Nazarbayev University, Astana, Kazakhstan; 2 Department of Anesthesiology, Intensive Care, and Pain Medicine, National Research Oncology Center, Astana, Kazakhstan; 3 School of Engineering and Digital Sciences, Nazarbayev University, Astana, Kazakhstan; 4 Department of Artificial Intelligence Convergence, College of Information Science, Hallym University, Chuncheon, Republic of Korea

**Keywords:** artificial intelligence, machine learning, neurophysiological signals, pain assessment, pain detection

## Abstract

**Introduction:**

Pain management remains a critical challenge in clinical settings. This scoping review studied the applications of artificial intelligence (AI) in pain medicine, particularly pain detection, prediction, and classification.

**Methods:**

A comprehensive search was conducted in PubMed, Scopus, and Cochrane Library databases up to July 2025.

**Results:**

Forty-seven relevant studies were included. The findings demonstrated that AI may have the potential for objective pain assessment using facial expressions, electroencephalography, neuroimaging, and physiological signals. Detection models achieved high accuracy, frequently exceeding 85%–90%. Predictive models showed efficient forecasting in pain sensitivity, pain evaluation after operation, and pain treatment response. Classification approaches also reported high performance. The results are hindered by several limitations: risk of overfitting, shortage of real-world experiments, homogeneous or small data samples, and methodological heterogeneity. Critical barriers for clinical implementation are ethical concerns and external validity.

**Conclusion:**

AI showed significant potential as a pain assessment and decision-support tool in pain medicine and research, especially for populations unable to self-report. It is recommended to use large, diverse datasets and real-world clinical validation to ensure the effective transitioning of artificial intelligence into healthcare systems.

## 1 Introduction

Pain is an unpleasant sensory and emotional experience associated with actual or potential tissue damage. It is subjective, shaped by biological, psychological, and social factors, and thus cannot be read off from only tissue damage. Pain is distinguished from nociception (the mechanism via which noxious stimuli are encoded) because nociception can occur without pain and pain without nociception (1). So, a signal that tracks noxious stimuli is not always a measure of the pain a person might experience. Pain is a major cause of disability. If left untreated, it can lead to negative personal and social outcomes, such as depression and work absenteeism. It also imposes unnecessary pressure on families and caregivers but also urges individuals to seek continuous medical help from many physicians, especially when appropriately pain controlled is not achieved (1, 2). Pain is usually detected, assessed, and monitored by communication with patients using history taking and different pain assessment scales. However, some patients, such as infants, Alzheimer's patients, intubated and mechanically ventilated patients, and unconscious patients, cannot self-report pain through oral or written communication. Therefore, it is challenging to assess pain in this group of patients and make a further diagnosis. Critically, the absence of self-report in such groups removes the reference standard for training and validation of automated systems. Defining a trustworthy ground truth — a patient's self-reported pain — is a central challenge for pain evaluation based on AI. Hence, other methods should be considered for pain assessment. In such cases, technological support can be helpful. However, despite advancements in medical technology, there is still a significant need for accurate pain detection and management. Recent progress in brain imaging techniques has resulted in neurocomputational models that utilize neurological signals to classify different pain states. These models analyze changes in the structure and function of the brains of chronic pain patients (3, 4). Electroencephalogram (EEG) data-based models have associated specific neuronal activities in the motor cortex with various pain states. EEG is a non-invasive, cost-effective, and widely available method for predicting brain function abnormalities and extracting markers of chronic pain (5).

Chronic pain is conventionally determined as pain persisting for more than three months. It is not an entirely sensory signal but a biopsychosocial condition. Nociceptive (biological) input interacts with affective, cognitive, and social processes. Artificial-intelligence (AI) systems have almost entirely worked with the biological dimension — electroencephalographic (2, 3, 6, 7), facial (8–11), neuroimaging (5, 12, 13), and physiological correlates of pain (14–16). This is because such signals can be measured and labelled. The psychosocial dimension, which determines how pain is experienced and reported, is largely unaddressed by current models. Therefore, multimodal systems combining several biological domains based on patient-reported and behavioral data are needed.

Chronic pain disrupts the adaptive integration of sensory and contextual processes and is associated with significant structural and functional changes in the brain. EEG has been shown to effectively track these changes. However, existing EEG studies on chronic pain-related cortical activity have yielded inconsistent results (1, 4). Some studies have reported increased theta and gamma oscillations in chronic pain patients, while others have observed reductions in alpha and beta power. Further research is needed to establish an efficient brain-based marker for chronic pain (7, 17).

Machine learning can effectively improve the pain-related neuroimaging analysis. It can uncover patterns in clinical and experimental data, providing valuable information for acquiring new knowledge and understanding the complexity of pain. Convolutional Neural Network (CNN), known for its success in computer vision and speech recognition, has shown potential for analyzing EEG signals due to its ability to handle large datasets and exploit hierarchical structures in natural signals (2). Several studies have explored the application of CNN in EEG analysis for various purposes, achieving impressive results (6). However, there is limited work on using CNN for EEG-based chronic pain detection, which is a challenging but important task in pain diagnosis and treatment. The goal of this scoping review was to synthesize and analyze the available evidence related to the application of AI in pain research and make relevant clinical and scientific recommendations.

The main families of AI used in the studies reviewed are defined here: Classical machine learning (e.g, random forests and logistic regression) analyzes predefined, hand-engineered features and is more effective on structured, tabular information (laboratory values or questionnaires). On the other hand, deep learning learns features from raw data via multi-layer neural networks. Its subtypes can be used with different signal types: convolutional neural networks (CNNs) can read images and image-like inputs (e.g., facial photographs, MRI, and spectrograms); recurrent networks with long short-term memory (LSTM) and bidirectional LSTM (BiLSTM) are used for time series such as EEG or physiological traces; graph neural networks (GNNs) can analyze data with an explicit relational structure, for instance, facial-landmark graphs; and transformer-based large language models (LLMs) can read text, which is a basis for conversational and patient-education programs. Multimodal/fusion models mix these streams. Since pain is multidimensional, integration of complementary signals is better matched to the construct in comparison to a single-signal approach.

## 2 Methods

**Figure 1:**
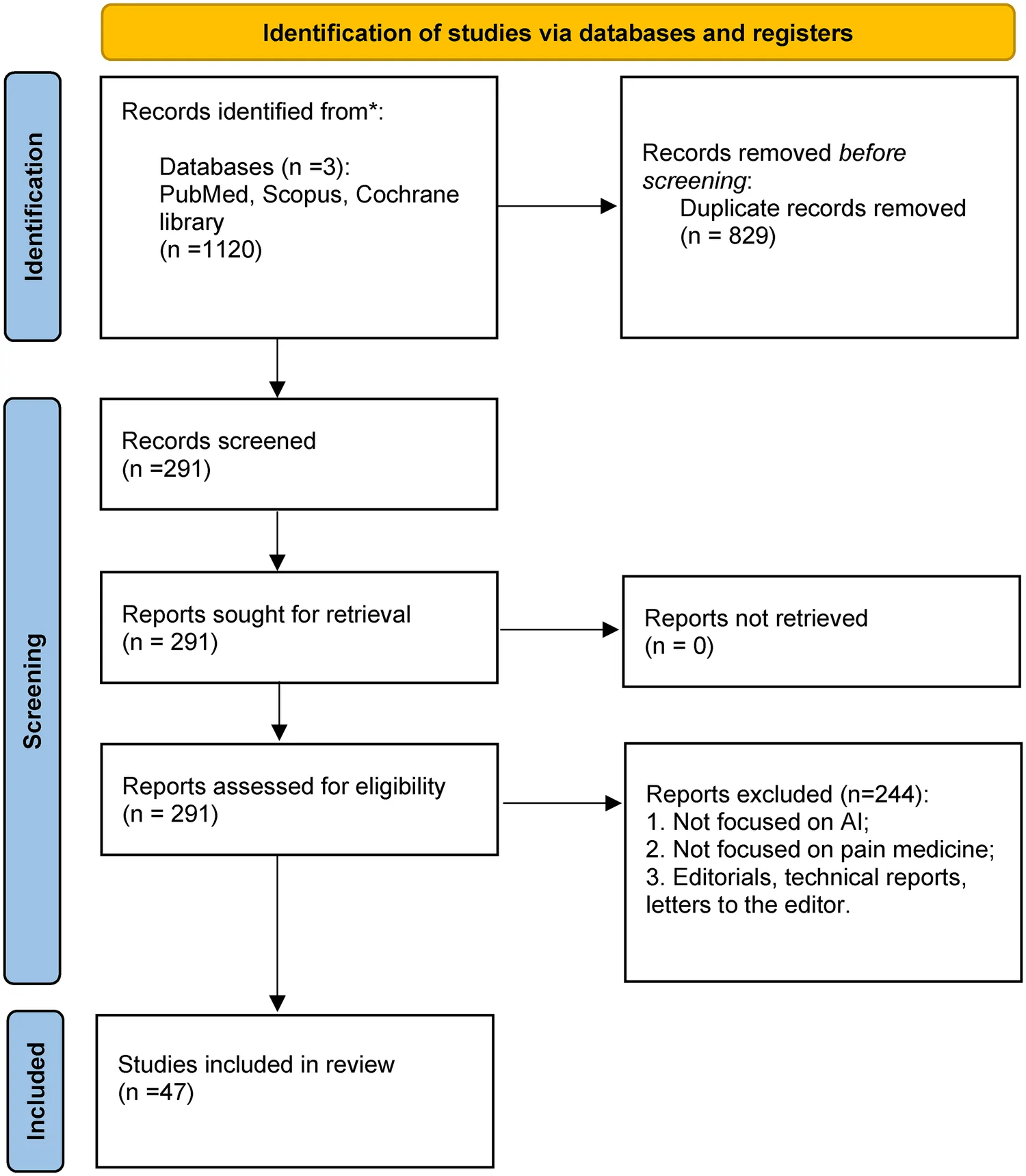
PRISMA 2020 flow diagram for systematic reviews that included searches of databases and registers only.

This scoping review was conducted in accordance with the Preferred Reporting Items for Systematic reviews and Meta-Analyses extension for Scoping Reviews (PRISMA-ScR) (18). The flow of records is summarized in the PRISMA 2020 flow diagram (Figure 1), and the completed PRISMA-ScR checklist is provided as Supplementary Material (Table 1).

**Table 1:** Artificial intelligence method and its purpose.

№	Author, country, year	Study type	AI algorithm, model, or method	Comparator (if any)	Purpose of AI	Benefits of AI	Risks of AI	Study conclusion
1	Duo Chen, Haihong et al., China, 2022 (2)	Model development study	A framework for detecting pain using Deep Convolutional Neural Networks (CNN) and EGG	No	In a sample of 10 chronic back pain patients, to identify pain states from resting to painful	System is potentially mobile, broadly available, cost-effective, and non-invasive for brain function abnormalities prediction and extraction of chronic pain's brain-based marker as EEG can register the brain functional processes' complex interplay more sensitive than others.	First attempt of deep CNN for the construction of objective EEG-based detector of chronic pain.	The receiver operating characteristic curve (AUC) higher than 0.80 was obtained when the proposed multi-layer network model was used for classifying EEG segments as “pain” and “non-pain.” Movement stimulation elevated AUC values in detection for the motor and occipital lobes. In comparison, the AUC for video stimulation was highest in the occipital lobe.
2	Fu-Jung Hsiao et al., Taiwan, 2021 (7)	Predictive model development study	K-nearest neighbor and SVM are among the machine learning models.	No	Predict and train feature selection with t-test from psychometric data and functional connectivity derived from psychometric measurements and EEG.	To classify the participants into low and high sensitivity groups, an accuracy of 86.2%–93.8% was provided by SVM, as 80% accuracy was obtained for the pain sensitivity.	The model has not been used on large scales other than low and high sensitivity.	For the use of pain perception's objective evaluation in clinical practice, SVM was used to predict and classify pain sensitivity.
3	Fengjie Wu et al., China, 2022 (6)	Model development study	A deep Attentive-Recurrent-Convolutional Neural Network (ARCNN)	No	With images of EEG ARCNN is proposed to study spatial-spectral-temporal representations and make them more conducive to subject-independent and multi-class scenarios.	Improvement over state-of-the-art and baseline methods in this field during a subject-independent scenario on a 4-level pain intensity assessment.	The model was used for the first time for pain assessment.	The model demonstrated the capability to obtain comparable images of EEG from different EEG caps' EEG time series as model has a large-scale dataset to merge EEG data from various sources.
4	Qi Han et al., China, 2021 (32)	Prognostic model development study	The linear discriminant analysis	No	To predict the intensity of postoperative pain with pre-surgery EEG recordings.	The algorithm is portable, fast, and simple, making it superior to others when its assumptions are met.	Small sample size problem, and not all discriminative information can be presented in the means of classes.	The level of acute postoperative pain was predicted by the LDA, which analyzed neural oscillations' features, showing a correlation coefficient of 0.84 for the relation of real and predicted pain intensities and 92.54% prediction accuracy.
5	Atsushi Kimura et al., Japan, 2021 (45)	Model development study	Support vector machine using the Radial Basis Function Kernel (RBFK)	To compare pain groups, Welch's *t*-test was applied.	To recognize hip pain during walking and divide it into pain quartiles.	During training, instead of the entire dataset, RBFK need store only the support vectors.	If the number of units is large, RBFK has greater difficulties because it is more sensitive to dimensionality.	Recognition accuracy of 79.6% was obtained during the subdivision of individual hip pain results recognized by the model into pain quartiles.
6	Jiahao Wang et al., China, 2020 (31)	Model development study	Autoencoder	Prediction accuracy of different models was compared (Logistic Regression (LR), Linear Discriminant Analysis (LDA), Support Vector Machine (SVM) and k-Nearest Neighbour (k-NN)).	To extract the features obtained from pain-related EEG signals	Autoencoding allows for reducing the use of data dimensionality and learning time, as well as compactness and speed in coding using backpropagation.	Correct operation requires a lot of resources in the form of a large amount of data, time, hyperparameter tuning, and model validation.	In classifying high-pain and low-pain EEG epochs, the proposed AE method is significantly more accurate. According to this study, the proposed deep learning methods and AE model can potentially be used to predict pain based on EEG.
7	Gomutbutra, et al., Thailand, 2022 (39)	Retrospective validation study	An automated facial analysis algorithm OpenFace was utilized to identify facial action units (FAUs).	No	To estimate the accuracy and practicality of the model based on the data from OpenFace© to categorize pain intensity in elderly Asian patients.	It may be suitable to augment gross triage tasks, such as supporting evidence of self-rating severity.	Higher misclassification in moderate pain than in obvious mild or severe pain. It may not be feasible to adopt the current automated pain severity classification model for critical decision-making, such as adjusting the dosage of opioid analgesics.	The accuracy of OpenFace© compared to human FACS coding is 90% for constrained images and 80% for everyday life images. However, the validity of OpenFace© in detecting a face in elderly individuals and dementia is still problematic. Higher misclassification in moderate pain than in obvious mild or severe pain. Retraining facial action unit algorithms, enhancing frame selection strategy, and adding pain-related functions may improve the accuracy and feasibility of the model.
8	Semwal and Londhe, India, 2021 (22)	Model development study	The hybrid model consisting of CNN and a BiLSTM model was used to extract spatial and temporal information from the head pose time series.	SVM, CNN-LSTM, CNN-RNN, Random Forest	The head pose-based pain detection model is compared with existing approaches.	It has been trained on a larger pain dataset Head movements provide information when there are no facial expressions.	Subjects in an uncontrolled environment may have movements that cause erroneous classification; In addition, there may be no head movements for low pain intensities.	Head movements provide important information related to pain behavior. The use of CNN-BiLSTM improves the performance of the model.
9	Kornprom Pikulkaew and Varin Chouvatut, Thailand, 2021 (43)	Model development study	Python 3.5.1 is used to build pain perception using deep learning algorithms learning. The UNBC Shoulder Pain Dataset is used to train pain-sensing.	No	To classify pain based on people's facial expressions and gauge its severity.	The proposed approach effectively handles circumstances for which 24-hour patient monitoring is insufficient.	It is proposed to include sequential images since the amount of pain between the initial pain and the pain is not balanced.	Deep learning methods achieved a 99% precision.
10	S Jerritta et al., India, 2022 (29)	Model development study	EMG and ECG signals were collected from the Biovid Heat Physiological Signal Database. The HRV signals have been derived from ECG signals and used for pain assessment. Daubechies wavelets of different orders (1 to 8) are used to perform the time-frequency transformation, after which a set of statistical features, energy features, and higher-order statistical features are extracted/ these features are fed into a K Nearest Neighbor (KNN) and a Regression Tree (RT) classifier to classify the pain levels.	No	To extract HRV signals from ECG signals using Pan Tompkins Algorithm, analyze HRV and EMG signals separately, and combine them in the time-frequency domain using the Daubechies wavelet for pain assessment.	To reduce the effects of noise (thermal and powerline) and other artifacts, these physiological signals are preprocessed through a Butterworth Bandpass and a notch filter.	In the present work, we have considered two physiological signals among the three physiological signals in the database (ii) a small group of subject's data has been used for train and testing the classifier to identify the pain (iii) only Daubechies wavelet functions are used for time-frequency transformation (iv) a simple set of statistical features are used to classify the pain levels, and (v) two simple nonlinear classifiers are used for pain classification.	The KNN classifier demonstrated superior accuracy in assessing no pain and high pain compared to the RT classifier. EMG signals provide more valuable information about pain compared to HRV signals.
11	Kelati et al., Sweden, Tunisia, Saudi Arabia, 2022 (16)	Model development study	A machine learning-based pain level classification system using data collected from facial electromyograms (EMG) is used A pain-associated facial electromyography (fEMG) features classification is performed by K-nearest neighbor (KNN)	No	To use the facial expression associated with pain amplified on the corrugator and zygomaticus EMG on the classification analysis, independent of a subject bias,	Considerable growth in classification accuracy is noticed when the subject matter from the features is omitted from the analysis And a lack of a subject bias.	The number K has an impact on the result; with a higher K, the classification performance result will be reduced. The zygomaticus is vague for pain indication, with no pain, neutral, and negative emotions.	The proposed classification algorithm has achieved a 99.4% accuracy for classifying the pain tolerance level from the baseline (P0 versus P4) without the influence of a subject bias. The facial zygomaticus EMG actions are associated with no pain. The corrugator is good for indicating when there is pain or no pain; however, the zygomaticus is vague with no pain, neutral, and negative emotions.
12	Morabit et al., France, 2022 (11)	Model development study	Pre-trained data-efficient image transformers with distillation (DeiT)	Convolutional Neural Networks (CNN)	to use fine-tuning of pre-trained data-efficient image transformers and distillation (Deit) for pain detection from facial expressions.	When using Transformers, the size of the dataset improves results.	The fact that the databases are not balanced is a potential cause of the difference obtained in accuracy.	The proposed architecture of a fine-tuned Deit for pain recognition exceeds the state-of-the-art methods in terms of accuracy.
13	Piri et al., Denmark, Sweden, 2022 (44)	Pilot study	Convolutional neural network	No	To quantify and compare PET/CT imaging data in order to identify potential nociceptive sources of low back pain.	AI-based segmentation improved processing speed and ensured 100% repeatability in PET/CT scan analysis.	Variability from different PET/CT scanners requires validation in larger, standardized studies.	AI-based processing enhances the feasibility of PET imaging for detecting inflammation and degenerative changes in low back pain.
14	Soin et al., USA, 2022 (33)	Pilot study	A decision tree machine learning software program	clinicians' diagnosis	To predict a spinal diagnosis using a machine learning approach.	A data set via machine learning may provide the treating physician with advice with respect to which therapy would be more successful on the basis of patient-reported data.	1) The generalizability of the study results is not definite due to the small study sample. 2) Since practitioner-assigned diagnoses were applied in machine learning, practitioner biases may have been included in the machine-learning algorithm.	The software was accurate at predicting practitioner-assigned diagnosis 72% of the time.
15	Kreiner & Viloria, Uruguay, 2022 (24)	Diagnostic model development study	A multilayer perceptron (MLP) that was trained using backpropagation algorithms	clinicians' diagnosis	To identify orofacial pain.	MLP can be potentially life-saving.	No data	The diagnostic accuracy of the artificial intelligence was greater than that of the general dental clinicians (*p* = .0072).
16	Gram et al., 2015, Denmark (30)	Predictive model development study	SVM	Conventional statistics	To classify on the basis of the EEG recorded before morphine administration, morphine responders and non-responders.	SVM classifies participants with a 72% accuracy rate when traditional group-based analysis proved insufficient.	There is a need for greater populations and more concentrated electrodes.	Separating responders and non-responders using machine learning was made possible by EEG data collected before treatment.
17	Jerritta et al., 2022, India (14)	Model development study	Mouth detection is done with the help of Object Detection using the Viola-Jones Algorithm machine learning KNN and Regression Tree Classifiers	No data	To analyze geometric features obtained from the mouth area.	The proposed classifiers are simple and require a limited number of hyperparameters to be tuned for achieving higher classification accuracy.	A few subjects, a limited number of facial features. Classifiers may not perform well if the feature size is larger, while considering a greater number of subjects.	The pain signals are identified with good accuracy of 95% - 97% using the KNN classifier.
18	Ghosh et al., 2023, India (37)	Model development study	Convolution neural network (CNN), statistical, and deep learning-based feature analysis	Vgg16, ResNet50, Inception-v3, Werner et al., Lucey et al.	to detect the face regions, transform facial images into features, and compute scores for pain intensities. The scores are used to assess the performance of the proposed system.	The proposed system has better performance than the other competing methods.	No data	The proposed system achieves 81.54% accuracy for UNBC 2-class problem, 81.33% accuracy for UNBC 3-class problem, and 74.59% accuracy for D2 2-class problems. The proposed system produces better results for the deep learning-based approach than for the statistical one.
19	Kristian et al., 2023, Indonesia (40)	Model development study	Autoencoders, Short-Time Fourier Transform	No data	Autoencoders were used to collect the features from the infant's face and voice spectrograms and produce the latent-vector. The latter was used to construct the cry detection and pain classification models.	CAE and VAE can recreate the data with a small SSIM loss value.	A dense latent-vector had decreased the model performance for both CAE and VAE. Multimodal feature requires extra resources. Limited data might have introduced bias.	The best model was the use of CAE without dense latent-vectors. Using voice features (dB-scaled spectrogram) generated a higher F1 score and accuracy than the facial features. A multimodal feature that used the dB-scaled spectrogram for audio representation and CAE for facial feature extraction produced high accuracy.
20	Patania et al., 2022, Italy (19)	Model development study	Graph Neural Network	state-of-the-art-models	To classify video-based pain.	No data	No data	The proposed method demonstrates effectiveness in comparison with others.
21	Prajod et al., Germany, 2022 (28)	Model development study	Explainable Artificial Intelligence techniques, Layer-wise Relevance Propa-gation	No	No data	A cross-dataset evaluation of both models is performed The LRP method is relatively robust to sanity Checks.	The clinical pain model doesn't perform well in cross-database evaluation.	The within-database accuracy of both models is similar to the results of other research papers. The clinical pain model produces better overall performance and recognition of pain images (85% vs. 66% in experimental). The performance of the clinical pain model is considerably lower in cross-database evaluation.
22	Hu et al., USA, 2019 (23)	Feasibility study	Experiments consisting of three steps that combine augmented reality (AR), neural network (NN)-based artificial intelligence, and optical neuroimaging (fNIRS)	No	To evaluate the applicability of the CLARAi - clinical augmented reality (AR) and artificial intelligence (AI) platform for real-time, objective pain localization and identification based on mobile neuroimaging.	By integrating up to a 10-second data history, the CLARAi model was able to extract information from the data's temporal sequence in addition to its spatial pattern.	The CLARAi model is still developing.	Based on the brain statuses in the data set, the framework anticipated when and where there would be physical discomfort and interactively showed neuroimaging data in real time.
23	Xu et al., USA, 2018 (41)	Model development study	AU codings per frame were retrieved for each 10-second video sample to produce a sequence of AUs, both automatically by the iMotions software and partially manually by a Facial Action Coding System-trained human.	With vs. without transfer learning model	To develop a transfer learning model to first link automated features to manual characteristics associated with discomfort, and then classify using the transmitted characteristics.	This made it possible to use information from a different domain to enhance classifier performance on the clinically important task of automatically differentiating pain levels in hospitals.	Due to insufficient data for developing an accurate mapping from all automatic AUs to all manual AUs, Principal Component Analysis dimensionality reduction was applied.	The transfer learning model outperformed the control model in terms of performance (AUC = 0.72 vs. AUC = 0.67).
24	Fontaine et al., France, 2022 (10)	Model development study	ResNet-18 convolutional neural network	AI vs. healthcare professionals	To recognize and categorize a large sample of patients' facial expressions based on the severity of their pain.	A deep learning AI algorithm can predict pain intensity on an 11-point numeric rating scale (NRS) with a mean inaccuracy of 2.4 points and distinguish between facial expressions linked to various levels of pain.	No data	Based on study of facial expressions, this method had sensitivity values of 89.7% for diagnosing moderate pain (4/10) and 77.5% for diagnosing severe pain (7/10).
25	Menchetti et al., USA, Turkey, 2019 (8)	Model development study	Automated Facial Expression Recognition (AFER) is a two-phase learning model that combines a deep neural network (DNN) and a Convolutional Neural Network (CNN)	Emotient vs. proposed VGGFace2	To create a deep learning-based end-to-end AFER that jointly detects all pain-related Action Units (AUs).	The UNBC dataset (UNBC+) was enhanced with data from the CK + dataset, which not only increased the number of positive instances for each class of AUs but also added non-pain-related AUs as negative examples to strengthen the system.	Due to randomization, training and test sets could have comparable image content.	8 out of 9 AUs associated with pain are monitored by an Emotient system, while the proposed approach performs better in terms of accuracy and F1-scores.
26	Lopez-Martinez et al., USA, Canada, 2019 (12)	Model development study	Multi-task learning (MTL), hierarchical Bayesian logistic regression (HBLR)	Single-task vs. multi-task learning	The development of individualized classifiers for the presence of pain was carried out using MTL. HBLR model was used to develop individualized logistic regression classifiers using time-frequency data obtained using the continuous wavelet transform.	It allows to train several non-identical tasks in parallel, and what is learned in one task can be transferred to another, just as tasks can use potential information that can be obtained from other classification tasks.	Small sample size, little participant variability, and a dearth of meta-data.	The system may differentiate between different pain states more effectively by customizing the machine learning models utilizing MTL.
27	Fabi et al., USA, 2022 (20)	Experimental study (algorithmic bias)	Machine learning (ML)	No	ML model that is taught to recognize facial action units associated with painful expressions evaluated the activation of these units in created faces.	Even though the developed CV model was mostly trained on one race, it does not exhibit a significant racial bias in the detection of pain-related AUs.	No data	The artificially created faces can be used to train machine learning models and also help to reduce the number of confounding variables.
28	Bargshady et al., Australia, 2020 (9)	Model development study	Enhanced joint hybrid using Convolutional Neural Networks coupled to a collaborative bidirectional long-term short-term neural network algorithm (EJH-CNN-BiLSTM)	SVM, CNN-LSTM, CNN-RNN vs. EJH-CNN-BiLSTM	An algorithm was developed to extract the most notable features, choose them, and categorize the pain degrees.	This approach allows for the automatic assessment of four levels of pain from facial expressions, and the process of feature extraction and classification algorithm occurs in one workflow, making it simple to deploy in a real-time online system.	CNNs have very poor classification accuracy when dealing with small datasets.	EJH-CNN-BiLSTM performs well in identifying pain, with average accuracy of 91.2% for the training set, 90% for the testing set, and 98.4% for AUC.
29	Bargshady et al., Australia, 2020 (38)	Model development study	Ensemble deep learning model (EDLM)	EDLM vs. a standard VGG-Face and one-stream LSTM model	EDLM was created to recognize the multi-classification level pain severity using the participant's video frame pictures.	EDLM model produces better classification in multi-class situations and has a lower qualifying error.	Not stated	The suggested framework outperforms the competing approaches when used in a multi-level pain detection database, producing feature classification accuracy above 89% and a receiver operating characteristic of 93%.
30	Huang et al., 2025, China (34)	Prognostic model development study	-Linear Discriminant Analysis (LDA) -Support Vector Machine (SVM) -Neural Network (NN)	A widely used clinical prognostic tool called the STarT Back Tool (SBT).	To predict the recurrence risk of chronic low back pain (CLBP) in patients over a 2-year period by utilizing multidimensional medical information.	-Superior predictive accuracy compared to existing tools like the STarT Back Tool (AUC 0.813 vs. 0.555);-Enables personalized risk stratification for better-targeted preventive and multimodal management;-Potentially reduces overtreatment of low-risk and undertreatment of high-risk patients, optimizing healthcare resource use;-Helps improve patient quality of life by mitigating adverse outcomes related to CLBP recurrence.	-Potential recall bias in follow-up data collection;-Some significant prognostic variables may not have been included, limiting comprehensiveness;-MRI use for prediction is cautioned against in patients without red flags or ineffective treatment due to the risk of unnecessary resource use and patient anxiety.	The AI-based multidimensional model significantly outperformed the SBT, achieving an AUC of 0.813 versus 0.555 for the SBT. The MLR-MDM also demonstrated better specificity (70.2% vs. 17.6%) with a slightly lower sensitivity than SBT (85.2% vs. 93.3%). Future external validations are needed to confirm the model's stability and performance.
31	He et al., 2025, China (46)	Retrospective study (diagnostic model)	Deep learning, specifically convolutional neural networks (CNNs).	2 comparator groups: Pure Human Group and AI-Assisted Human Group	To assist in the diagnosis of lumbar disc herniation (LDH) by analyzing MRI images to detect specific features such as herniations, protrusion size, and nerve root compression.	-Improves diagnostic accuracy and reduces decision-making time;-Provides objective and consistent image assessments, reducing misdiagnosis risk.	-False positives may lead to unnecessary interventions and patient anxiety;False negatives can delay critical treatments and worsen outcomes;-Discrepancies between AI and human diagnoses highlight challenges in interpretation;-Ethical issues (patient data privacy and the need for clinician oversight).	AI-assisted diagnosis for lumbar disc herniation significantly improves accuracy and decision speed compared to traditional methods. Future research should focus on larger, diverse datasets, longitudinal studies, and standardized AI implementation protocols.
32	Mao et al., 2025, China (47)	Diagnostic model development study	The YOLOv10 (You Only Look Once version 10) algorithm, a state-of-the-art real-time target detection algorithm.	Human experts (specifically senior oral radiologists)	To achieve automated diagnosis and classification of temporomandibular joint (TMJ) degenerative joint disease (DJD) on cone beam computed tomography (CBCT) images.	-High diagnostic accuracy and efficiency, outperforming human experts in speed and precision; Ability to detect and classify multiple typical radiographic signs simultaneously;-Reduction in diagnostic complexity and workload for dentists lacking specialized training.	-Variability in accuracy depending on dataset size and AI methods used;-Potential for misclassification or partial accuracy, especially with images showing multiple signs;-Dependence on high-quality, annotated datasets for training.	AI has the potential to quickly review CBCT images and assist in the accurate and rapid diagnosis and classification of TMJ DJD.
33	Choi et al., 2025, Republic of Korea, Australia, USA (48)	Diagnostic model development study	Pre-trained convolutional neural network (CNN) architectures commonly used for image classification, including GoogleNet, ResNet, and VGGNet.	8 orofacial pain specialists.	To create a screening and diagnostic tool for degenerative joint disease (DJD) of the temporomandibular joint (TMJ) by integrating TMJ panoramic radiography images with joint noise data (both clinician-detected crepitus and patient-reported joint noise).	-Improved Diagnostic Accuracy; -Early Detection and Better Patient Care; -Comprehensive Diagnostic Tool; -Support for Non-Specialist Clinicians;-Interpretability.	-Lower Performance Compared to Some Previous Studies; Limited Data Modalities and Clinical Context; -Incomplete Clinical Noise Data; Dataset and Study Design Limitations.	The study supports the integration of AI with multi-modal diagnostic data as a valuable advancement in DJD diagnosis, highlighting its potential to surpass traditional diagnostic methods while recognizing the need for ongoing refinement and validation.
34	De Araujo et al., 2024, Brazil (42)	Validation study (diagnostic tool)	PAINe AI assistant using natural language processing (NLP) and classification logic	Validated clinical pain questionnaire	To differentiate odontogenic pain from temporomandibular disorder (TMD) pain.	High accuracy, rapid initial screening, improves accessibility for preliminary dental pain triage	Not tested on real patients, Chatbot responses may be over-confident without clinical oversight.	The PAINe AI virtual assistant showed high agreement with the standard screening tool. This may support dental pain differentiation in preliminary assessment.
35	Liawrungrueang et al., 2024, Thailand (49)	Model development and validation study (diagnostic)	Deep learning model based on a convolutional neural network (CNN) architecture specifically the “You Only Look Once” (YOLO) framework.	Expert radiologist annotations	To accurately detect and grade lumbar intervertebral disc degeneration (IDD) using MRI images according to the Pfirrmann grading system.	-Enhances diagnostic accuracy and reliability for lumbar intervertebral disc degeneration (IDD) grading; -Reduces diagnostic variability among clinicians; -Saves time and resources by automating the grading process; -Supports clinical decision-making and treatment planning.	-Potential overfitting leading to reduced generalizability to diverse populations; -Dataset bias due to limited patient demographics and anonymous imaging devices; -Reliance on the subjective Pfirrmann grading system may introduce interpretation variability.	The developed artificial intelligence (AI)-based deep learning model demonstrates high accuracy, sensitivity, and specificity in detecting and grading lumbar intervertebral disc degeneration (IDD) using the Pfirrmann grading system based on MRI scans [accuracy (up to 97%), sensitivity, specificity, F1 score, prediction error, and ROC-AUC (ranging from 92% to 97%) across training, testing, and external validation datasets]. There is a need for more diverse datasets to validate the model's applicability across different demographic groups, the subjectivity involved in the Pfirrmann grading system, and the necessity for prospective clinical trials to evaluate the real-world impact of AI integration in diagnostic workflows.
36	Sada et al.,2024, Kosovo, Australia (26)	Validation study (construct validity)	Supervised machine learning with independent training and validation datasets; Automated recognition and analysis of an infant's facial expressions.	Observer-Administered Visual Analog Scale (ObsVAS): 100-mm line where 0 mm represents no pain or distress and 100 mm represents the worst possible pain or distress.	To automatically recognize and analyze six facial action units (AUs) — such as brow lowering, nose wrinkling, and lip corner depression — which are validated indicators of pain in infants.	-Automated, objective, and rapid pain assessment; -Reduced subjectivity compared to traditional observer-based pain scales; -High accuracy with an area under the curve (AUC) of around 0.96, strong diagnostic performance.	-Still requires user attention; -Clinical judgment is still necessary. -Limited evidence about overall clinical utility beyond this study; -Requires parent training.	The AI-based PainChek Infant tool can effectively inform parents' decision-making regarding pain identification and management in infants after circumcision surgery.
37	Ah-Yan et al.,2024, Canada (50)	Qualitative observational study	Large language models (LLMs)	ChatGPT by OpenAI vs. Copilot by Microsoft	-To enhance patient counseling and self-management of low back pain. -To provide personalized medical information and clinical advice to patients, helping them better understand and manage LBP.	-Supports self-management of LBP by patients; -Reduce healthcare costs, and time of healthcare specialists; -Available	-Inaccurate, incomplete, or biased information; -Legal and ethical concerns regarding the use of AI-generated content in medicine; - Generic and not sufficiently tailored to specific clinical contexts.	-Validity scores: ChatGPT 3.33, Copilot 3.18 -Safety scores: ChatGPT 3.19, Copilot 3.13 -Usefulness scores: ChatGPT 3.60, Copilot 3.57. The inclusion of specific clinical context in the questions did not significantly affect the quality (validity, safety, usefulness) of the responses. Recommended for cautious use by patients.
38	C Areias et al., 2024, USA, Portugal (35)	Prognostic model development study	Recurrent Neural Network (RNN) and Light Gradient Boosting Machine (LightGBM)**,** a tree-based binary classifier)	RNN vs. LightGBM	To identify early those patients who are less likely to achieve meaningful pain reduction (nonresponders).	-Enables continuous, real-time prediction of patient pain response throughout treatment; -Improves model performance over time through dynamic data updates; -Identifies key predictive features.	-Potential biases in predictive accuracy related to demographic or socioeconomic factors, challenge in equity in model use; -The AÌs reliance on data quality and completeness.	The RNN achieved an ROC-AUC of 0.70 (95% CI 0.65-0.71) and LightGBM an ROC-AUC of 0.71 (95% CI 0.67–0.72) at session 7; Both models showed high specificity when precision was prioritized. Pain during exercise, baseline severity (disability and mental distress), motivation barriers, and exercise performance were among the most predictive of response.
39	Cascella et al.,2024, Italy, India (21)	Feasibility study	YOLOv8 (You Only Look Once, version 8), based on Darknet-53, a robust convolutional neural network (CNN).	No	Real-time pain assessment by analyzing facial expressions using computer vision techniques.	-Provides objective and quantifiable pain assessment; -Enables real-time detection of pain expressions; -Automates facial action unit recognition, reducing the need for expert manual scoring.	-Dataset limitations affecting generalizability to broader real-world contexts; -Model performance decreased under uncontrolled conditions. -Potential false positives and false negatives.	The model trained using YOLOv8 showed consistent improvement during training, with a precision of 0.818, a recall of 0.807, and a mean Average Precision (mAP) of 0.874 by epoch 10. The precision for pain detection was 0.868, and for no-pain instances, 0.919, indicating high accuracy in distinguishing pain and non-pain facial expressions.
40	Muhaimil et al., 2024, India (13)	Predictive model development study	Machine learning (ML) classifiers: Random Forest, AdaBoost, Decision Tree, Logistic Regression, KNN; Deep learning (DL) algorithms: GoogleNet, ResNet (ResNet18, ResNet50).	Random Forest; AdaBoost; Decision Tree; Logistic Regression; K-Nearest Neighbors (KNN); GoogleNet; ResNet (including ResNet18 and ResNet50).	To improve the prediction of low back pain (LBP) by analyzing T2-weighted MRI images of the lumbar spine.	-Objective, reproducible, and efficient extraction of quantitative features from medical images; -Machine learning (ML) and deep learning (DL) models improve the prediction accuracy of low back pain (LBP) using MRI; -AI can identify subtle abnormalities not visible to the naked eye.	-Overdiagnosis and misinterpretation without careful clinical context; -AI models may require larger sample sizes for improved robustness and reliability; -Data quality, model generalizability.	Among ML classifiers, Random Forest and AdaBoost models showed the most reliable performance in predicting LBP. Random Forest achieved area under the curve (AUC) values ranging from 0.83 to 0.88 across all lumbar vertebrae, with the highest AUC of 0.92 at the L5-S1 intervertebral disc (IVD). AdaBoost showed AUC values between 0.82 and 0.90, with the highest AUC of 0.97 at the L5-S1 intervertebral disc. Decision tree models showed moderate performance (AUC 0.65 to 0.76), and logistic regression performed well, particularly at the L5 vertebra with an AUC of 0.82 and good performance at other levels. Among DL models, GoogleNet outperformed others, achieving an accuracy of 0.85 after 30 epochs, followed by ResNet-18 with 0.84 accuracy.
41	Granviken et al., 2025, Norway (51)	Cluster randomized controlled trial	Case-Based Reasoning (CBR)	Usual physiotherapy care	To learn from past patient cases to suggest personalized treatment options for new patients thereby facilitating pain management for musculoskeletal disorders.	-Facilitates personalized patient care by learning from previous cases using case-based reasoning (CBR); -Provides an overview of biological, psychological, and social factors.	-Limited treatment fidelity; -Limiting effectiveness.	There were no significant differences in global perceived effect (GPE) between patients treated by physiotherapists with access to the CDSS and those receiving usual care; For the Patient-Specific Functional Scale (PSFS), a larger proportion of patients in the control group (usual care) reported clinically important improvement compared with the intervention group using the CDSS (70% vs. 60%), making its clinical importance uncertain.
42	Snene et al., 2024, Switzerland (27)	Feasibility study	Light Gradient-Boosting Machine (LGBM) classifier	Conventional clinical pain scales	To predict pain intensity and frequency among elderly patients with dementia based on physiological signals such as electrodermal activity (EDA), skin temperature, heart rate (HR), and motion data collected via wearables.	-Real-time, non-invasive, and continuous pain monitoring in patients with dementia who cannot communicate effectively; Improved accuracy of pain assessment.	-Limited holistic assessment; -Challenges in accurately annotating and contextualizing data, which may affect the model's reliability; -Pain may not be fully captured through physiological signals alone since it is a subjective sensation.	The Light Gradient-Boosting Machine (LGBM) classifier showed optimal performance in recall and precision for pain detection. Electrodermal activity (EDA), skin temperature, and mobility data were identified as the most appropriate physiological signals for detecting pain.
43	Zapata-Caballero et al., 2025, Mexico (52)	Evaluation study (LLM responses)	ChatGPT 3.5, a language-processing artificial intelligence chatbot developed by OpenAI	No	To provide informative answers to patient-generated queries about chronic pelvic pain (CPP).	-Quick and informative answers; -Scores highly in accuracy, clarity, and relevance when answering queries.	-Lack completeness and consistency, particularly regarding support groups and further reading resources; - Inaccurate or nonexistent information, such as fake website links.	The AI responses scored highest in accuracy, clarity, and relevance, with median scores of 3 (out of 3) in these domains. Completeness and consistency were rated lower, with median scores of 2, indicating some answers failed to fully address aspects of the questions, and variability was observed in responses to repeated queries.
44	Cheema et al., 2025 (36)	Retrospective analysis (prognostic ML)	Bootstrap random forest supervised machine learning Traditional statistical models	Traditional statistical models	To identify prognostic variables associated with physical-therapy outcomes in chronic low back pain.	Higher predictive accuracy. Ability to analyze high-dimensional healthcare data and detect variables not previously recognized.	Limited interpretability; inability to provide effect sizes or causal inference; potential selection bias	Machine learning can predict prognostic factors and support clinical prognosis, treatment planning, and research design.
45	Temel et al., 2025, Turkey (53)	Comparative evaluation study (LLM vs. specialists)	ChatGPT, version 4o of the Generative Pretrained Transformer (GPT) language model developed by OpenAI.	Assessments by two physical medicine and rehabilitation specialists, each with 10 years of experience.	To accurately and consistently identify and represent lumbar radicular pain (LRP) patterns (L4, L5, and S1 radiculopathies) on an anatomical model.	AI can be beneficial in specific tasks such as image analysis, improving diagnostic accuracy and reducing detection errors.	Inaccuracies and inconsistencies in AI outputs can lead to misdiagnosis or inappropriate treatment. Widespread use without sufficient validation and regulation may jeopardize patient safety and outcome.	Pain patterns of L4, L5, and S1 lumbar radiculopathy (LRP) generated by ChatGPT-4o were statistically significantly different from those of experienced specialists (*P* < 0.001 for all comparisons). ChatGPT's follow-up markings taken two weeks later were also significantly different from its initial responses, indicating inconsistency over time.
46	Shahid et al., 2024, Pakistan, Turkey (15)	Predictive model development study	Random Forest (RF); Gradient Boosted Trees (GBT); Multilayer Perceptron (MLP) Neural Network; Radial Basis Function (RBF) Neural Network.	Comparisons among models themselves	To analyze various attributes such as uric acid, C-reactive protein, creatinine, and other blood parameters to accurately classify and anticipate joint pain.	-AI models provide high accuracy (up to 97–98%) in predicting joint pain from biochemical data, aiding early diagnosis; -Early detection through AI helps prevent serious orthopedic complications like arthritis and osteoarthritis.	-Slower processing times despite good prediction performance; -AI requires well-established computerized medical systems; -Accuracy depends on the quality and quantity of data.	The Random Forest decision model outperformed other models with an accuracy of approximately 97% and minimal errors in predicting joint pain based on biochemical parameters. The Gradient Boosted model also achieved the same accuracy, but with higher relative errors. The multilayer perceptron (MLP) performed better than the radial basis function-neural network (RB-NN), achieving around 98% accuracy for predicting joint pain. AI models are recommended for clinical use to improve early diagnosis and treatment interventions.
47	Giordano et al., 2024, Austria, Germany (25)	Comparative validation study	FaceReader 9 software, an AI-based facial recognition and emotion detection tool.	Assessment of neonatal pain by a panel of 46 healthcare professionals. They have used visual analogue scale (VAS).	To identify and quantify facial action units (AUs) associated with pain responses in neonates. To generate metrics of arousal and valence reflecting the intensity and emotional quality of the infants' facial expressions in response to stimuli of varying pain intensities.	-Provides objective, accurate, and real-time detection and analysis of neonatal facial expressions related to pain; -Capable of distinguishing between different intensities of painful stimuli through metrics like arousal and valence.	-Results may not directly apply to preterm infants or those in intensive care units, limited generalizability; -Some data loss occurred due to failed face detection from movement or suboptimal angles.	AI showed a significant correlation with pain evaluations conducted by 46 healthcare professionals using a visual analogue scale (VAS). This validates the AI system's ability to reflect human assessments of neonatal pain. Pearson correlation between arousal and expert pain assessments: r = 0.84 (*p* < 0.001). Pearson correlation between valence and expert pain assessments: r = 0.86 (*p* < 0.001). Correlations between type of stimulus and expert pain ratings: r = 0.84 (*p* < 0.001).

To identify relevant publications, we systematically searched PubMed, Scopus, and the Cochrane Library from the inception up to July 2025. The search terms used included the following keywords:

- (“artificial intelligence” OR “machine learning” OR “deep learning”) AND “pain”

The search was limited to English-language publications. We also reviewed the references of relevant articles to identify additional appropriate studies. The inclusion criteria for studies were as follows: (1) using AI algorithms; (2) management of acute or chronic pain; and (3) types of studies: observational studies, randomized controlled trials. Studies were excluded if they were letters to the editor, editorials, conference abstracts, systematic reviews with meta-analyses, consensus statements, or guidelines unrelated to the research topic. Two reviewers independently conducted the literature search and screened the titles/abstracts, with disagreements resolved by the third author. Data extraction included information such as author names, publication year, gender, study goals (primary, secondary), sample size, diagnosis, surgery, AI algorithm, model or method, comparator, purpose of AI, benefits of AI, challenges of AI, and author conclusions. In this study, deep learning referred to studies that utilized deep neural networks (e.g., convolutional neural networks) as the primary algorithm, while other studies were categorized as classical machine learning.

## 3 Results

**Figure 2:**
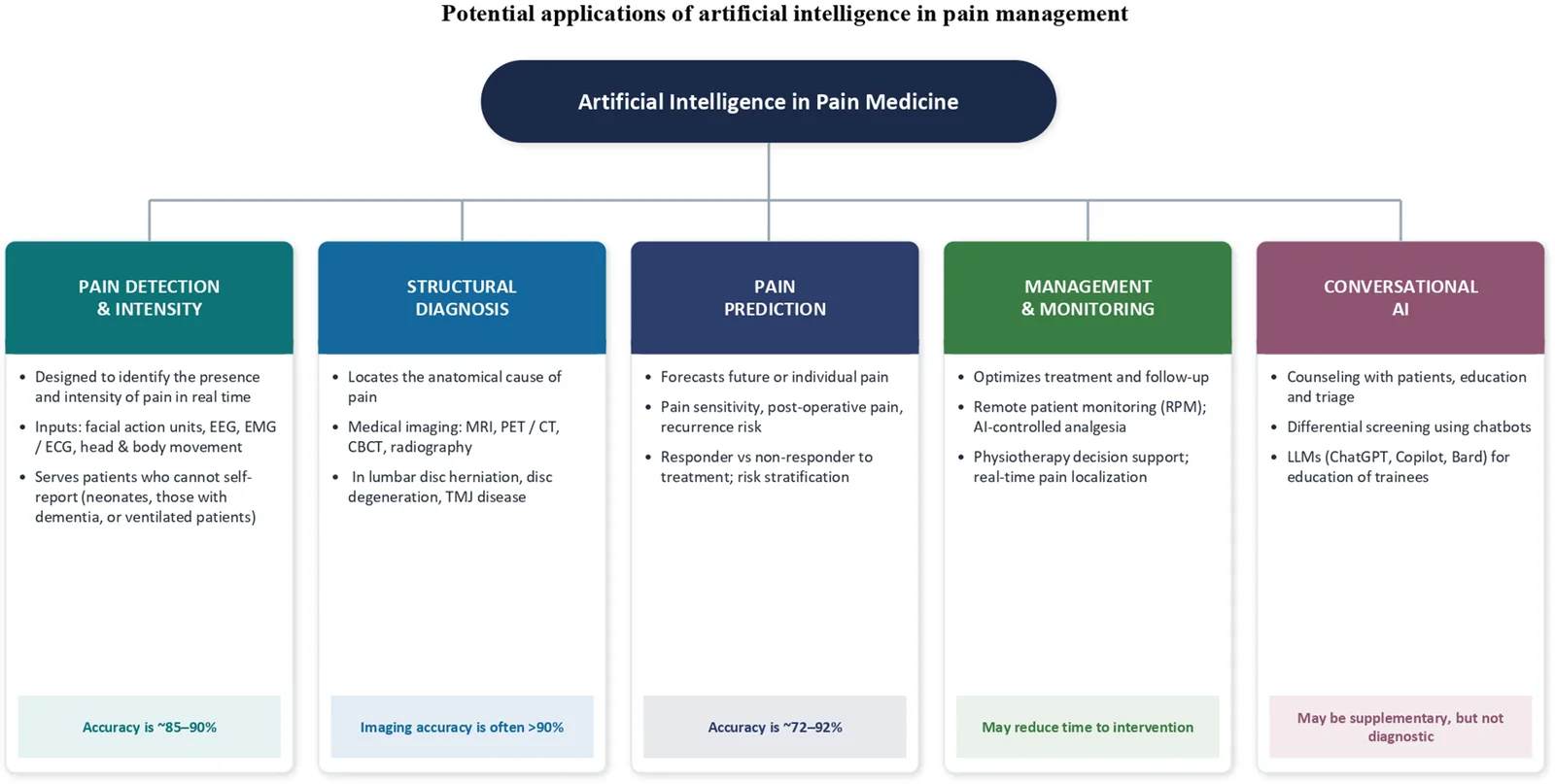
Potential applications of artificial intelligence in pain management.

**Figure 3:**
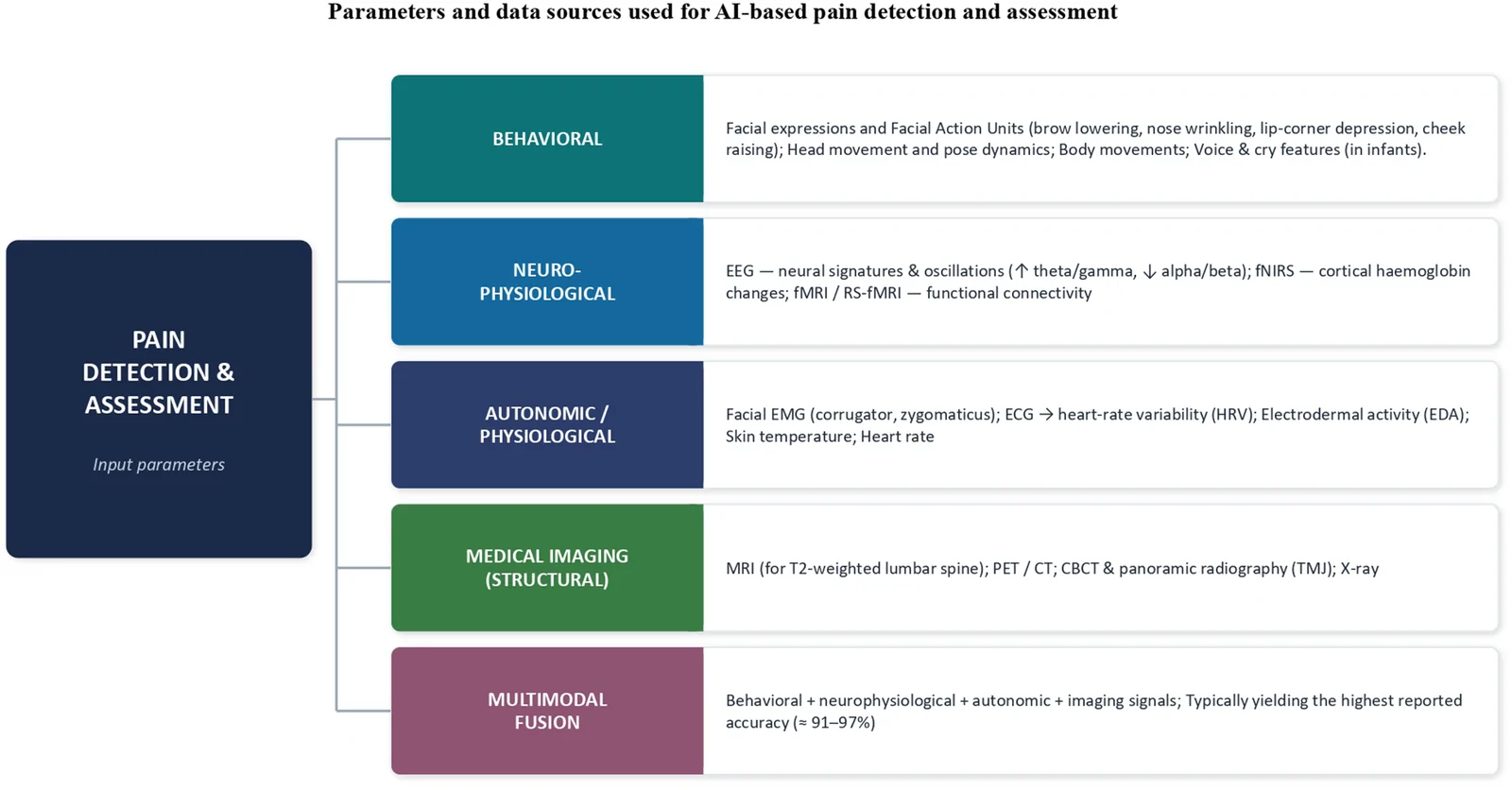
Parameters and data sources used for AI-based pain detection and assessment.

The initial search identified 1120 articles. After removing duplicates, 291 records were screened for eligibility. After careful review, 47 articles were considered suitable for inclusion (2, 6–16, 19–53) (Figure 1). The characteristics of included studies are described in the Supplementary material (Table 2). Table 1 describes in detail the AI method used and its purpose. AI algorithms and models are described in Table 2. Potential applications of artificial intelligence in pain management are summarized in Figure 2, and Parameters and data sources used for AI-based pain detection and assessment are summarized in Figure 3.

**Table 2:** Description of AI algorithms (based on data from included studies).

**Main AI algorithms**	
1	Deep Convolutional Neural Networks (CNN) for EEG: CNNs are primarily used for image processing, but are also applied to time-series data like EEG. The convolutional layers help identify local patterns within the EEG data, making them effective for pain detection.
2	K-nearest neighbor and SVM: Both are traditional ML algorithms. KNN classifies data based on the majority class of its ‘k' nearest data points, while SVM finds the optimal hyperplane that separates data of different classes.
3	Capsule Network Model: This is an evolution of CNN. Capsule networks aim to better capture spatial hierarchies between features, potentially leading to better performance and fewer data requirements.
4	Deep Attentive-Recurrent-Convolutional Neural Network (ARCNN): Combines attention mechanisms with recurrent and convolutional layers, optimizing feature extraction from time-series EEG data.
5	Linear Discriminant Analysis: A method used to find a linear combination of features that best separates two or more classes in a dataset.
6	Combinatorial CNN Structure: CNNs with various combinations of architectures to optimize feature extraction.
7	sLORETA Source Localization: A technique to identify the location of neural sources from EEG data.
8	Support Vector Machine with RBFK: SVM using a Radial Basis Function Kernel, which can handle non-linear data.
9	OpenFace for Facial Action Units: An open-source tool for facial landmark detection, which is used to detect and analyze facial expressions.
10	CNN and BiLSTM Hybrid Model: Combines the spatial feature extraction capabilities of CNN with the sequential pattern learning of BiLSTM.
11	KNN and Regression Tree for Pain Levels: Using KNN and decision trees to classify different levels of pain.
12	Multilayer Perceptron (MLP) with Backpropagation: A basic neural network architecture trained with the backpropagation algorithm.
13	BIRCH Algorithm: Used for hierarchical clustering, usually for large datasets.
14	Object Detection with Viola-Jones Algorithm: A rapid object detection method, particularly known for face detection.
15	Graph Neural Network: Used for data structured as graphs, capturing relationships between nodes.
16	Explainable AI Techniques: Methods to make AI models' decisions understandable to humans.
17	Layer-wise Relevance Propagation: A method to explain neural network decisions by attributing relevance to each input feature.
18	Crystalace & CrystalFeel with Word2Vec: Emotion analytics algorithms that might use Word2Vec for semantic understanding of language.
19	ResNet-18 CNN: A deep CNN architecture with 18 layers known for its “residual” connections that help in training.
20	AFER: Combines DNN and CNN to analyze facial expressions in a two-phase learning process.
21	Multi-task Learning (MTL) and HBLR: MTL trains on multiple related tasks simultaneously, while HBLR is a Bayesian approach to logistic regression.
22	EJH-CNN-BiLSTM: An enhanced model combining CNNs and BiLSTMs for feature extraction and sequence learning.
23	Deep Learning, Dlib, and ResNet Pre-model: Combining deep learning techniques with the Dlib library (for facial detection) and pre-trained ResNet models.
24	Ensemble Deep Learning Model (EDLM): Combines multiple deep learning models to optimize performance.

Complementary models essential for enhancing the AI algorithms	
1	Bootstrap Random Forest – An ensemble method in machine learning that generates data by the bootstrap resampling technique.
2	Gradient boosting family – Boosted-tree ensembles like XGBoost, AdaBoost, that may outperform single trees.
3	LightGBM (Light Gradient Boosting Machine) – A gradient boosting framework for machine learning.
4	Logistic regression – Standard linear classifier.
5	Autoencoders – An artificial neural network that is trained by minimizing reconstruction loss.
6	DeiT (data-efficient image transformers) – More efficiently trained transformer architectures for image classification.
7	YOLO family – Real-time object detection models used for face.
8	GoogleNet/ Inception-v3/VGG (pretrained CNNs) – CNNs designed for image classification trained on the ImageNet dataset.
9	Case-Based Reasoning – An approach where new problems are solved by using similar past cases.
10	Graph Neural Networks (GNN) – Deep learning that can infer from graph-structured data. Can handle irregular data structures.

### 3.1 AI-assisted pain detection

Sixteen studies included in this review explored AI-driven approaches for better pain detection. Among them, six focused on enhancing pain detection through facial expression analysis and one on head movement. In 2019, Menchetti developed an Automated Facial Expression Recognition (AFER) system for detecting pain-related Action Units (AUs) in non-verbal patients. Compared to the Emotient system, AFER demonstrated superior accuracy and F1-scores, successfully identifying 8 out of 9 pain-associated AUs (8). Bargshady (2020) introduced an enhanced deep neural network that couples a convolutional neural network with a bidirectional long short-term memory (BiLSTM) network (the EJH-CNN-BiLSTM model) for multi-class pain intensity detection from facial expression images, achieving an average accuracy of 91.2% on the training set, 90% on the testing set, and 98.4% AUC (9). Later, Patania et al. (2022) proposed a graph neural network (GNN) that utilized the graph structure of facial fiducial points. On the BioVid Heat Pain database (the model was given to distinguish no-pain vs. highest-intensity pain; the fairness was tested by leave-one-subject-out cross-validation), it surpassed standard models, reaching 73.2% accuracy. This figure reflects genuine (stimulus-evoked pain) rather than acted expression and was considered a state-of-the-art result for that benchmark at the time (19). In the same year, Fontaine et al. (2022) trained a DL system (ResNet-18) on 2,810 images from 1,189 patients, achieving high sensitivity (89.7%) for detecting severe pain (≥4/10) and outperforming nurses, who identified pain with a lower sensitivity (44.9%) (10). Fabi et al. (2022) investigated racial bias in facial action-unit (FAU) detection. A model that was predominantly trained on European faces analyzed synthetic European and African faces in the study. This bias was not shown to be uniform. Although European faces were favored, several action units were readily detected on African faces too. This shows that the visual detection of individual muscle activation may vary with skin tone or race-dependent features. The authors concluded that greater exposure to own-race faces and perceptual detection ability do not explain the racial bias in pain recognition (20). Recently, in a feasibility study, Cascella et al. (2024) evaluated YOLOv8 object detector on a custom pain/no-pain dataset using facial expression images (*n* = 500). The precision showed 0.868, however, a small balanced subset may not reflect clinical diversity (21). Notably, complementing facial cues, Semwal and Londhe (2021) previously proposed an automated pain detection method using head movement dynamics, leveraging a CNN-BiLSTM network to learn spatial and temporal dependencies. Evaluated on the UNBC-McMaster dataset, this approach outperformed facial expression-based methods, suggesting that head pose analysis may be a more reliable indicator of pain and a promising alternative for automated pain assessment (22).

Three studies explored AI-based methods in orofacial pain. In 2019, Hu et al. developed CLARAi, a mobile neuroimaging-based augmented reality and AI framework for real-time pain detection and localization. Functional near-infrared spectroscopy was used to measure cortical activity in 21 patients experiencing tooth pain, with a three-layer neural network achieving 80.37% accuracy for pain classification and a six-layer convolutional neural network reaching 74.23% accuracy for pain localization (23). Later, Kreiner and Viloria (2022) introduced a multilayer perceptron neural network for diagnosing orofacial pain and temporomandibular disorders. Their model outperformed 12 general dental clinicians (*p* = .0072), particularly in detecting non-dental and referred pain (24). In the same year, Jerritta et al. leveraged machine learning for pain detection based on facial geometry, focusing on mouth area features. Using the Biovid Heat Pain database, KNN and Regression Tree classifiers achieved 95%–98% accuracy, with KNN proving superior. Notably, geometric and ratio-based features were the most effective for pain assessment (14).

Four studies focused on automated pain detection in particular groups or sensors. Lopez-Martinez et al. (2019) utilized functional near-infrared spectroscopy (fNIRS) signals from 43 healthy participants to detect pain using Bayesian hierarchical modeling with wavelet features. Their model identified pain presence with 69% accuracy, based on cortical hemoglobin concentration changes (12). Detection of procedural pain in neonates with intensity discrimination was analyzed by Giordano et al. (2024). The study analyzed an AI facial-analysis system (FaceReader 9, “Baby Face” model). The results correlated strongly with experts' VAS (Visual analogue scale) (25). Post-surgical pain assessment was conducted by Sada et al. (2024). The AI-based PainChek Infant tool enabled parents to make informed decisions about whether to apply intervention or not. This led to reduced pain in 94.5% of assessments (26). Snene et al. (2024) tested the feasibility of continuous AI tracking of pain intensity (Empatica E4 wristbands) in patients with dementia. LightGBM algorithm was found to have the highest accuracy (0.74) (27).

Two studies aimed to optimize the data inputs for more accurate pain detection. Prajod et al. (2022) used Explainable AI to analyze differences between pain detection models trained on experimental (BioVid) and clinical (UNBC) pain datasets. While both convolutional neural networks achieved similar within-dataset accuracy, the clinical pain model demonstrated superior generalizability, recognizing pain with 85% accuracy compared to 66% for the experimental model (28). In the same year, Jerritta (2022 b) developed a machine learning-based pain detection method utilizing EMG and HRV signals from the Biovid Heat Physiological Signal Database. After preprocessing with Butterworth and notch filters, Daubechies wavelets were applied for feature extraction. As a result, KNN outperformed Regression Trees in detecting pain levels, with EMG signals emerging as the most effective for pain recognition. Based on the presented findings, advanced AI-driven methods have the potential to improve the accuracy and reliability of automated pain detection, particularly across different age and racial groups (29).

Summarizing all the studies, two main observations can be highlighted. Firstly, facial expression is considered the most mature modality. Deep models that are trained on facial action units either matched or exceeded clinical expectations, with Fontaine et al. reaching 89.7% sensitivity for severe pain in comparison to 44.9% for bedside nurses (10) and the network of Kreiner and Viloria, which outperformed twelve dentists (24). The second observation is that the accuracy is significantly dependent on the data source and not only on the algorithm alone. For example, Prajod et al. demonstrated that models trained on clinical pain from the UNBC-McMaster archive generalise better than those trained on experimentally induced pain from BioVid (85% vs. 66%) (28). This may explain why accuracy in the section may fluctuate from roughly 73% on BioVid (19) to the mid-90s on UNBC-derived data (14). A recurring caution is fairness, as Fabi et al. showed that the detectability of pain-related facial cues may vary by itself with skin tone (20), thus, a model validated on one population cannot be referred to another.

### 3.2 AI-assisted pain prediction

Ten studies have proposed AI-driven methodologies to improve pain prediction. Four studies were conducted across different modalities. In 2015, Gram et al. applied ML to EEG data recorded during cold pain stimulation to predict individual morphine analgesic response, achieving 72% accuracy using SVM models trained on spectral features (30). In 2020, Wang et al. proposed an autoencoder model based on convolutional neural networks (EEGNet) for feature extraction of pain-related EEG signals, which had a superior accuracy of 74.6 ± 11.2% in pain level prediction compared to conventional methods (31). The following year, Hsiao et al. utilized multimodal neurophysiological and psychometric signatures to predict individual heat pain sensitivity, achieving up to 80% prediction accuracy using SVM models trained on resting-state EEG features (7). Also in 2021, Han et al. achieved a notable increase in precision, attaining 92.54% accuracy and a correlation of 0.84 between real and predicted pain levels by using pre-surgery EEG recordings and an LDA model to predict postoperative pain intensity (32).

Four studies were conducted in condition-specific states. Soin et al. (2022) used machine learning algorithms to predict diagnoses related to spinal pain by analyzing 85 specific data points. The decision tree software achieved a 72% accuracy rate in predicting the diagnosis based on the reported symptoms. The study suggests that AI-driven methods have the potential to enhance pain prediction in clinical settings, particularly in analyzing low-level visual information (33). Huang et al. (2025) also studied low back pain and found that Multivariate Logistic Regression (MLR) within a Multidimensional Model (MDM) framework achieved AUC = 0.813 for future recurrence risk forecasting and outperformed the standard STarT BACK Tool (34). Muhaimil et al. (2024) specifically used T2-weighted MRI of the lumbar spine to predict low back pain, where the best deep learning was GoogLeNet with 0.85 accuracy (13). Shahid et al. (2024) used age and sex together with serum biochemistry, including uric acid, C-reactive protein, creatinine, and other blood and liver-function parameters, to predict joint pain by comparing several models. The best-performing model at 97.44% accuracy was a random forest, which combined bootstrap sampling, random feature selection at each split, and tree pruning. Uric acid was identified as the strongest single predictor (15).

Two studies reviewed AI for prediction after treatment. One study, Areias et al. (2024), used Light Gradient Boosting Machine (LightGBM) and Recurrent Neural Network (RNN) to predict treatment response using time-series data such as range of motion (ROM), exercise performance, psychological scores, and social deprivation indices. Both models achieved similar performance with an AUC of 0.70 (35). Another study by Cheema et al. (2025) studied chronic low back pain to predict which patients are associated with better or worse pain-related rehabilitation outcomes. The study applied Bootstrap Random Forest (RF) supervised machine learning, where the Random Forest model achieved an overall 75.6% predictive accuracy (36).

Based on prediction studies, two distinctive targets can be traced. First is the forecasting of intrinsic pain sensitivity or analgesic response, which analyses the neurophysiology. For instance, Gram et al. forecasted morphine response from pre-stimulus EEG (about 72%) (30), Hsiao et al. predicted heat pain sensitivity (80%) (7), and Han et al. reached 92.5% for acute post-operative pain (32). The second target implies prediction of clinical outcomes like chronicity, recovery, and joint pain based on routinely collected information (15, 33–36). More modest models are clinically applicable and externally oriented — Areias et al. reported an AUC near 0.70 for a musculoskeletal recovery model (35). In contrast, the prediction value is explicit, where it outperforms the existing tool, as in Huang et al., whose multidimensional model was more effective than the STarT Back screening tool (0.813 vs. 0.555) (34). Nevertheless, prediction is considered the least represented domain due to the gap between controlled and externally validated settings.

### 3.3 AI-assisted pain classification

Eighteen studies have introduced AI-driven methodologies to improve pain classification. Eight studies advanced pain classification using facial expression analysis with ML and DL models. Morabit et al. developed a data-efficient image transformers and distillation (DeiT) model for binary pain recognition, achieving 84.15% accuracy on the UNBC-McMaster dataset and 72.11% on the BioVid dataset, outperforming the pre-trained GoogleNet model (11). Similarly, Ghosh et al. introduced a sentiment analysis system to classify pain intensity levels, reaching 82.54% and 83.71% accuracy for 2- and 3-class UNBC-McMaster databases and 75.67% on the 2D Face-set with Pain-expression dataset (37). Bargshady et al. introduced an ensemble deep learning model (EDLM) for pain level classification, integrating a three-stream hybrid deep neural network. This model achieved 89% accuracy and an AUC of 0.93 on the Multimodal Intensity Pain (MIntPain) database, surpassing other deep learning methods for multi-level pain detection (38). Meanwhile, Gomutbutra et al. applied five ML models to classify pain in chronic pain patients using FAUs, with SVM incorporating FAUs and gender achieving the highest accuracy of 58% in the Asian elderly population (39). Kristian et al. developed a multimodal ML-based system for infant pain classification using facial expressions and voice features, with ensemble models outperforming single-modality approaches based on F1 scores (40). Xu et al. improved pediatric pain classification by applying transfer learning to map automatically detected FAUs from iMotions software to manually coded AUs, increasing the AUC from 0.67 to 0.72 (41). Kelati et al. explored facial electromyograms (fEMG) for pain level classification, with KNN achieving 99.4% accuracy, indicating that EMG signals from the corrugator and zygomaticus muscles reliably differentiate pain from non-pain conditions (16). Lastly, de Araujo et al. (2024) used ChatGPT-4 and built-in Python/Streamlit to classify odontogenic and temporomandibular pain. AI achieved 86% accuracy (42).

Eight studies advanced pain classification focusing on specific conditions. In 2021, Pikulkaew et al. applied DL techniques to detect shoulder pain using sequential facial images, achieving an impressive accuracy of 98.75% for no-pain and 95.05% for pain classification, compared to doctor diagnoses. However, the classification accuracy for initial pain levels dropped to 75.49%, primarily due to an imbalanced dataset. Despite this, the technique performed well in scenarios where continuous monitoring of patients for over 24 h is not feasible (43). Similarly, Piri et al. (2022) applied AI-based classification methods to PET/CT scan data, distinguishing regions of interest based on fluorodeoxyglucose and sodium fluoride uptake. The results indicated heightened tracer uptake in the facet joints and intervertebral discs of individuals with low back pain, suggesting inflammation and degenerative changes as potential sources of nociceptive pain (44). Kimura et al. (2021) developed a wearable EEG system to assess pain during movement, addressing the limitations of resting-state evaluations. Using a single-electrode device and machine learning, the system successfully classified hip pain with 79.6% accuracy (45). Chen et al. (2022) utilized deep convolutional neural networks (CNN) to classify pain states from EEG signals in 10 chronic back pain patients, achieving an AUC of 0.83 ± 0.09 during movement stimulation and 0.81 ± 0.15 during video stimulation, surpassing existing methods in accuracy (2). He et al. (2025) focused on a deep learning Convolutional Neural Network (CNN) model in patients with lumbar disc herniation (LDH). This model achieved 93.4% accuracy, which can automatically detect LDH (46). Mao et al. (2025) aimed to use a YOLOv10 object-detection–style deep network for the detection of temporomandibular joint degenerative disease, with 98% accuracy reported for the classification of radiographic signs in an image (47). The same condition detection was studied by Choi et al. (2025), where ResNet-18/50/101, VGG-16/19, and GoogLeNet outperformed eight orofacial pain specialists on a 200-image reader set (48). A Convolutional Neural Network using the YOLO (You Only Look Once) architecture (CNN-YOLO) was studied by Liawrungrueang et al. (2024) for the detection of lumbar intervertebral disc degeneration. The model achieved 97% accuracy in training for implicit grading of discogenic back-pain severity (49).

Two studies advanced pain classification across different contexts. In 2021, Hsiao et al. utilized multimodal neurophysiological and psychometric signatures to classify individual heat pain sensitivity, achieving up to 93.8% classification accuracy using support vector machine (SVM) models trained on resting-state EEG features (7). Later, Wu et al. (2022) presented a deep learning-based approach for objective pain intensity assessment in non-verbal patients, utilizing multi-channel EEG signals to learn invariant spatial-spectral-temporal representations. By employing an Attentive-Recurrent-Convolutional Neural Network (ARCNN), the model achieved 92% accuracy in pain classification in a subject-independent scenario (6). All of the above studies provide evidence that AI-based methods could significantly refine pain classification workflows, particularly through neuroimaging, with the potential for real-time applications.

The classification studies all show the same dividing line. Behavioral classifiers of pain expression based on facial, EEG, or EMG signals show wide variation in accuracy (58%–99%). Kelati et al. reached 99.4% on EMG (16), while Gomutbutra et al. achieved 58% on a clinical facial action unit sample (39). This tracks the dataset quality and class balance. In contrast, structural classifiers that may label the anatomical lesion from MRI, CT, PET, or CBCT are more consistent, reaching clustering at 93%–98% (46–49). Such an observation may, however, be misleading for pain assessment because degenerative changes are commonly asymptomatic. So, grading disc degeneration or temporomandibular pathology is a classifying structure, not the pain experience itself.

### 3.4 Remote technologies

Three articles reported AI accuracy for remote patient monitoring, patient education, and counseling. Ah-Yan et al. (2024) studied pain management support ChatGPT (GPT-3.5) and Copilot (GPT-4) for the generation of safe, valid, and useful advice for low-back pain. On a 4-point Likert scale, AI provided 3.33 for validity, 3.19 for safety, and 3.60 for usefulness. This demonstrated good performance (50). Granviken et al. (2025) described how AI-driven personalized decision support can be used to manage musculoskeletal pain using multilevel mixed logistic models. The results were insignificant [measured by Odds ratio (OR)] (51). Zapata-Caballero et al. (2025) evaluated patient education by ChatGPT 3.5 by asking common patient questions. It achieved the highest total scores in lifestyle and misconceptions (14/15), lowest in support groups 9/15 and further reading 10/15 (52).

One study evaluated the reliability of generative AI outputs. Temel et al. (2025) assessed whether ChatGPT-4o can consistently and accurately draw L4/L5/S1 lumbar radicular pain patterns. The results indicated poor intra-model consistency (53).

### 3.5 Medical conditions

AI has been used for pain management in a wide variety of diseases and conditions, such as acute postoperative pain (10, 32), chronic pain (2, 39), hip pain (45), low back pain (including lumbar radiculopathy, post-laminectomy syndrome, sacroiliitis, lumbar disc herniation, lumbar intervertebral disc degeneration, and degenerative lumbar spine diseases) (13, 33–36, 44, 46, 49, 50, 53), shoulder pain (9, 28, 37, 38, 43), temporomandibular disorders (TMD) and orofacial pain (24, 42, 47, 48), odontogenic pain (42), hypersensitive pain (23), joint pain (15), chronic pelvic pain (52), pain associated with dementia and neonatal infant procedures (25, 26, 40). Additionally, AI was used to assess pain sensitivity, anesthesia depth, and post-operative pain.

### 3.6 Tasks of AI in pain medicine and research

The studies were clustered into the following main thematic groups:

1. Automatic identification of the presence of pain or its intensity: This is the biggest group that utilized EEG-based neural signatures and oscillations, facial expressions and action units (AUs), physiological signals from EMG and ECG, video-based data, and data based on multimodal behavior. This group of AI algorithms allows unbiased real-time pain recognition with high accuracy.
2. Diagnosis of structural causes of pain: This group identifies the pathology of pain based on MRI, PET, and CT images. Such systems allow for increasing diagnostic accuracy, speed, and clinical decision-making.
3. Prediction of future pain: This group supports treatment planning and stratification of risks in predicting chronic pain, responder vs. non-responder classification, and response to therapies.
4. Management and monitoring: This group aims to improve clinical procedures such as analgesia, remote patient monitoring, physiotherapy, and neuroimaging of pain localization.
5. Conversational AI: This group expands AI as a tool for patient counseling, differential screening, and education in pain medicine.

### 3.7 Benefits and challenges of AI

Across the included studies tabulated, AI demonstrated a potential to measure pain objectively and non-invasively. For instance, EEG-based algorithms can detect pain via brain signatures with high sensitivity in comparison to traditional methods. Machine learning and deep learning can consistently provide high accuracy in classification and prediction. There is 80% accuracy for pain sensitivity prediction, and 90% accuracy in post-operative and facial expression-based detection. Likewise, imaging-based and multimodal systems have shown more than 90% diagnostic accuracy. AI can also be efficient in terms of computational performance. For example, autoencoders can decrease training time, kernel-based methods can minimize memory storage, and cross-validation can improve reliability and reduce overfitting. Additionally, lightweight models like linear discriminant analysis are suitable for clinical environments by being fast, portable, and easily deployable. Another benefit of AI is that it can extract data from raw signals that may be difficult to capture with traditional methods. CNNs, transformers, and hybrid architectures can learn to detect pain from EEG, video, and physiological data. The performance may be further improved by combining neuroimaging, facial expressions, and physiological signals. In cases where self-reporting is unavailable (infants, unconsciousness, and cognitive impairment), AI can still evaluate pain based on crying, facial expressions, or physiological monitoring. In addition, AI helps clinical decision-making by providing real-time monitoring and guidance. Overall, AI's main strength is that it can reduce bias and standardize the pain assessment. It may remove observer and racial biases to allow fairer assessment.

Despite the benefits, the included studies consistently report the challenges that can constrain AI's clinical implementation. The first challenge is the lack of generalizability due to small sample sizes and limited population diversity. Many models were trained on controlled and homogeneous data. Another challenge is the issue of scalability. Deep learning approaches' effectiveness could have been decreased due to small datasets, which can lead to overfitting. Next, multimodal systems, cross-validation, and training duration require resources that might be costly and unfeasible for clinical settings. There can also be specific challenges: in the case of facial expression analysis, there can be an overlap with other emotional states. Misclassification rates, even low, can significantly affect the decision-making in medication dosing and critical care, making AI not yet suitable in high-risk settings. Head-movement-based models can be erroneous in the case of minimal head movements in patients. Finally, it should be noted that many models are experimental and newly proposed.

## 4 Discussion

This review highlighted the recent results from experiments and observational studies of artificial intelligence (AI) applications in pain medicine. Overall, the results demonstrated consistent performance of AI—specifically deep learning architectures—on pain detection, prediction, and classification (6, 10, 32). Deep learning achieved high accuracy in controlled settings for objective pain evaluation (2, 9, 38). Nevertheless, the evidence revealed important limitations and challenges.

Beyond a study-by-study account, the included studies can be mapped onto the five functional roles described in section 3.6. The most populated role is the automatic identification of the presence or intensity of pain drawn from EEG, physiological signals, and facial expressions (2, 6–10, 16, 29). This may suggest that accurate pain detection may require integrated behavioral, multidimensional, and neurophysiological representation. The next role is diagnosis of the structural cause of pain. It is mostly based on imaging models (44, 46, 47, 49). The third role is relatively rare, which is pain forecasting for stratification and treatment planning (32, 34–36). Management and monitoring are the fourth role that covers remote tools and systems for decision support (27, 51). Finally, the fifth role is conversational AI used for education and counselling (50, 52). Such AI is commonly built on large language models.

This mapping of five roles uncovers six critical knowledge gaps that hinder clinical implementation. First is the problem of the ground truth against which an AI system trains and judges. The label is a self-report scale, like VAS or NRS, if a patient can communicate. As mentioned in the introduction, the populations of sedated, intubated, neonates, or those with dementia may not self-report, and studies use a surrogate. Two surrogates are mostly used: expert observation and the noxious stimulus. Expert observation utilizes validated behavioral scales. It can be labels assigned by the clinician or by an observational VAS scale (25–27). The noxious stimulus can be used itself in the calibrated heat of the BioVid database, for example. In this case, a controlled stimulus is applied to measure the body's involuntary reaction. These two surrogates possess distinctive cons. The model may inherit subjectivity and inter-rater reliability from a clinician, thus imitating rather than improving the judgement. Stimulus-linked models may conflate nociceptive input with perceived pain, which means they may not always capture pain correctly. This creates a paradox where the ground truth is the weakest in the populations that require automated assessment the most. Thus, reported accuracies in this population should be interpreted together with the reference standard used quality.

The second gap is the clinical relevance of structural imaging. Models that grade disc degeneration, detect herniation, or classify degenerative or temporomandibular disease showed one of the highest accuracies (13, 46–49). However, structural findings have only a loose correlation with actual experienced pain. This is due to the fact that the same degenerative changes may also appear in pain-free people. Therefore, these models are automated assistance for locating the anatomical source of pain. The models complement and do not duplicate the behavioural and neurophysiological detection work. They are not measures of pain intensity.

The third gap involves ethical concerns that accuracy figures may not capture. Three issues recur. Firstly, algorithmic bias: as mentioned before, Fabi et al. depicted that the detectability of related to pain facial cues differs with skin tone (20). So, a model trained on a demographically limited dataset might systematically produce errors in under-represented groups, which might lead to inequitable care and subjective assessment. Second is transparency and accountability, as many high-performing models are not transparent, which complicates analgesic decisions by an automated estimate. Third, governance of data and confidentiality: facial, neural, and physiological pain data are sensitive. The collection and reuse require safeguards: standardised, FAIR-compliant common data elements and repositories are needed. These considerations suggest AI as a decision-support adjunct with clinical supervision and without full autonomy.

The fourth gap is the underrepresentation of predictive and management models, as an imbalance in the literature can be traced. Pain identification looks developed and heavily studied, whereas prediction and management in real-world settings remain underdeveloped.

The fifth gap is the omission of psychosocial dimensions, where current AI systems only operate within the biological dimension (electroencephalographic, facial, neuroimaging, and physiological indicators). Cognitive and social processes that shape pain experience remain unaddressed.

The sixth gap is the translation to real-world settings. The heterogeneity of methods (evaluation metrics and datasets), small, homogeneous sample size, and generalizability issues (facial recognition system) preclude the synthesis of performance outcomes. Only a few studies assess the AI systems on real patients or clinical workflows. Nevertheless, encouraging experimental results with high accuracy show that AI can work as a decision-support tool to provide the subjective and context-dependent information for clinicians.

Therefore, the following directions for future research are recommended:

Research should be transitioned from retrospective analyses of controlled datasets to prospective clinical trials in order to test the external validity. Next, in order to address the psychosocial dimensions, researchers should design multimodal systems to integrate biological, patient-reported, and behavioral data. To refine the reference standards for vulnerable, non-verbal populations, future studies need to favor multi-rater standards in comparison to single-surrogate labels and adjust AI prediction to clinical outcomes, such as analgesic demands. To overcome methodological heterogeneity, researchers can standardize FAIR-compliant common data elements and repositories for uniform classification evaluation. To reduce errors in underrepresented groups, transparent decision-support models under clinical supervision and rigorously tested across varied demographics should be prioritized. Finally, to integrate conversational AI, further investigation of large language models' safety and factual accuracy should be directed to ensure that AI patient education supplements and does not displace clinicians.

## 5 Conclusion

To conclude, our scoping review highlights the clinical potential of AI to improve pain medicine through unbiased and automated assessment. By utilizing advanced machine learning and deep learning architectures (CNNs and LSTMs) to scrutinize neurophysiological, physiological, and behavioral biomarkers, these models may consistently achieve high accuracy—exceeding 85%–90%—offering a critical diagnostic method for vulnerable and non-verbal populations. However, systemic obstacles, including methodological heterogeneity, small and homogeneous samples, high risks of overfitting in models, and the omission of psychosocial factors, restrict immediate implementation. Technological transition into active healthcare structures might demand a paradigm shift toward multimodal fusion networks trained on expansive and demographically varied datasets and with rigorous, clinical validation.

## Data Availability

The original contributions presented in the study are included in the article/Supplementary Material, further inquiries can be directed to the corresponding author.
