# Supplementary material for: Potential applications of artificial intelligence in pain management: a scoping review
Source: Front Pain Res (Lausanne). 2026 Aug 26;7:1871943. doi: 10.3389/fpain.2026.1871943 (PMC13553931; doi:10.3389/fpain.2026.1871943)
Supplement: Supplementary file 1 [file Table1.docx]

**Preferred Reporting Items for Systematic reviews and Meta-Analyses extension for Scoping Reviews (PRISMA-ScR) Checklist**

| **SECTION** | **ITEM** | **PRISMA-ScR CHECKLIST ITEM** | **REPORTED ON PAGE #** |
| --- | --- | --- | --- |
| **TITLE** | | | |
| Title | 1 | Identify the report as a scoping review. | Page 1 (the title identifies the report as a scoping review). |
| **ABSTRACT** | | | |
| Structured summary | 2 | Provide a structured summary that includes (as applicable): background, objectives, eligibility criteria, sources of evidence, charting methods, results, and conclusions that relate to the review questions and objectives. | Page 2 (structured abstract: Introduction, Methods, Results, and Conclusion, with keywords). |
| **INTRODUCTION** | | | |
| Rationale | 3 | Describe the rationale for the review in the context of what is already known. Explain why the review questions/objectives lend themselves to a scoping review approach. | Pages 3–5 (Introduction). |
| Objectives | 4 | Provide an explicit statement of the questions and objectives being addressed with reference to their key elements (e.g., population or participants, concepts, and context) or other relevant key elements used to conceptualize the review questions and/or objectives. | Page 4 (explicit aim of the scoping review stated at the end of the Introduction; population, concept, and context defined throughout Pages 3–5). |
| **METHODS** | | | |
| Protocol and registration | 5 | Indicate whether a review protocol exists; state if and where it can be accessed (e.g., a Web address); and if available, provide registration information, including the registration number. | No review protocol was registered. The review was conducted and reported in accordance with PRISMA-ScR (stated on Page 6). |
| Eligibility criteria | 6 | Specify characteristics of the sources of evidence used as eligibility criteria (e.g., years considered, language, and publication status), and provide a rationale. | Page 6 (eligibility/inclusion and exclusion criteria, and study types). |
| Information sources* | 7 | Describe all information sources in the search (e.g., databases with dates of coverage and contact with authors to identify additional sources), as well as the date the most recent search was executed. | Page 6 (PubMed, Scopus, and the Cochrane Library, searched from inception to July 2025; reference lists of relevant articles also screened). |
| Search | 8 | Present the full electronic search strategy for at least 1 database, including any limits used, such that it could be repeated. | Page 6 (full electronic search string with all search terms). |
| Selection of sources of evidence† | 9 | State the process for selecting sources of evidence (i.e., screening and eligibility) included in the scoping review. | Pages 6–7 (titles/abstracts screened independently by two reviewers, disagreements resolved by a third author, Page 6; selection process shown in the PRISMA flow diagram, Figure 1, Page 7). |
| Data charting process‡ | 10 | Describe the methods of charting data from the included sources of evidence (e.g., calibrated forms or forms that have been tested by the team before their use, and whether data charting was done independently or in duplicate) and any processes for obtaining and confirming data from investigators. | Page 6 (data-extraction/charting variables listed; extraction performed by two reviewers). |
| Data items | 11 | List and define all variables for which data were sought and any assumptions and simplifications made. | Page 6 (all charted data items listed; deep learning vs. classical machine-learning definitions provided). |
| Critical appraisal of individual sources of evidence§ | 12 | If done, provide a rationale for conducting a critical appraisal of included sources of evidence; describe the methods used and how this information was used in any data synthesis (if appropriate). | Not performed. |
| Synthesis of results | 13 | Describe the methods of handling and summarizing the data that were charted. | Page 6 (study categorisation approach described); narrative/descriptive synthesis presented in the Results, Pages 7–18. |
| **RESULTS** | | | |
| Selection of sources of evidence | 14 | Give numbers of sources of evidence screened, assessed for eligibility, and included in the review, with reasons for exclusions at each stage, ideally using a flow diagram. | Page 7 (numbers of records identified [1120], screened [291], and included [47], with reasons for exclusion summarised in the PRISMA flow diagram, Figure 1). |
| Characteristics of sources of evidence | 15 | For each source of evidence, present characteristics for which data were charted and provide the citations. | Page 7; full characteristics and citations of each included source are provided in Table 2 (Supplementary material) and Table 2. |
| Critical appraisal within sources of evidence | 16 | If done, present data on critical appraisal of included sources of evidence (see item 12). | Not applicable. |
| Results of individual sources of evidence | 17 | For each included source of evidence, present the relevant data that were charted that relate to the review questions and objectives. | Table 2 (Supplementary material) and Table 2 and Results Sections 3.1–3.7 (Pages 7–18). |
| Synthesis of results | 18 | Summarize and/or present the charting results as they relate to the review questions and objectives. | Results Sections 3.1–3.7 (Pages 7–18), including the mapping onto five functional roles (Page 18) and Figures 2 and 3. |
| **DISCUSSION** | | | |
| Summary of evidence | 19 | Summarize the main results (including an overview of concepts, themes, and types of evidence available), link to the review questions and objectives, and consider the relevance to key groups. | Pages 18–21 (Discussion: overview of concepts, themes, and types of evidence, linked to the review aim and key groups). |
| Limitations | 20 | Discuss the limitations of the scoping review process. | Page 20 (Discussion of limitations: methodological heterogeneity, small/homogeneous samples, overfitting, and limited real-world/external validation). |
| Conclusions | 21 | Provide a general interpretation of the results with respect to the review questions and objectives, as well as potential implications and/or next steps. | Page 21 (general interpretation and implications/next steps at the end of the Discussion and Conclusion); abstract Conclusion, Page 2. |
| **FUNDING** | | | |
| Funding | 22 | Describe sources of funding for the included sources of evidence, as well as sources of funding for the scoping review. Describe the role of the funders of the scoping review. | Page 22 (review supported by Nazarbayev University Faculty Development Competitive Research Grant No. SOM2024005). Funding of the included studies was not separately reported. |

JBI = Joanna Briggs Institute; PRISMA-ScR = Preferred Reporting Items for Systematic reviews and Meta-Analyses extension for Scoping Reviews.

* Where *sources of evidence* (see second footnote) are compiled from, such as bibliographic databases, social media platforms, and Web sites.

† A more inclusive/heterogeneous term used to account for the different types of evidence or data sources (e.g., quantitative and/or qualitative research, expert opinion, and policy documents) that may be eligible in a scoping review as opposed to only studies. This is not to be confused with *information sources* (see first footnote).

‡ The frameworks by Arksey and O’Malley (6) and Levac and colleagues (7) and the JBI guidance (4, 5) refer to the process of data extraction in a scoping review as data charting*.*

§ The process of systematically examining research evidence to assess its validity, results, and relevance before using it to inform a decision. This term is used for items 12 and 19 instead of "risk of bias" (which is more applicable to systematic reviews of interventions) to include and acknowledge the various sources of evidence that may be used in a scoping review (e.g., quantitative and/or qualitative research, expert opinion, and policy document).

*From:* Tricco AC, Lillie E, Zarin W, O'Brien KK, Colquhoun H, Levac D, et al. PRISMA Extension for Scoping Reviews (PRISMAScR): Checklist and Explanation. Ann Intern Med. 2018;169:467–473. [doi: 10.7326/M18-0850](http://annals.org/aim/fullarticle/2700389/prisma-extension-scoping-reviews-prisma-scr-checklist-explanation).
