# Supplementary material for: Potential applications of artificial intelligence in pain management: a scoping review
Source: Front Pain Res (Lausanne). 2026 Aug 26;7:1871943. doi: 10.3389/fpain.2026.1871943 (PMC13553931; doi:10.3389/fpain.2026.1871943)
Supplement: Supplementary file 2 [file Table2.docx]

**Table 2.** Study and cohort information

| **№** | **Author, country, year** | **Study type**  **(For example, randomized control study, cohort study, case-control study, etc.)** | **Gender**  **(% male)** | **Study goals (primary, secondary)** | **Sample size** | **Diagnosis** | **Surgery** | **Author conclusions** |
| --- | --- | --- | --- | --- | --- | --- | --- | --- |
| 1. | Duo Chen, Haihong et al., China, 2021 | Cross-sectional study | No data | To identify chronic pain states by extracting neural signatures from EEG signals. | 10 volunteers, 4 with back pain and 6 with lower-back pain patients were recruited. | Chronic pain | No | In order to understand chronic pain and brain activity better, new EEG paradigms and methodologies can be applied. |
| 2. | Fu-Jung Hsiao et al., Taiwan, 2021 | Case-control study | 42.4% | Predicting individual heat pain sensitivities from the heat pain threshold (HPT) using the training dataset and the prediction dataset with high accuracy. | The experiment involved 85 right-handed people with normal MRI of the brain, not taking daily medications, without pain disorders during the anamnesis, and without distinctive pain during the year. | Heat pain sensitivity | No | The pain sensitivity’s underlying signatures could be detected in resting-state EEG data by intrinsic functional connectivity and neural oscillations analysis. For the use of pain perception’s objective evaluation in clinical practice, SVM was used to predict and classify pain sensitivity. |
| 3. | Fengjie Wu et al., China, 2022 | Experimental study | 70.8% | To study the invariant representation obtained by signals of the EEG and show its use in the classification of pain. | A total of 24 patients, 7 females and 17 males, 21 average age, without any illness. background, participated in the study | Pain intensity assessment | No | EEG-based classification tasks can be performed with the proposed approach, as the task of pain classification shows potential for use in the future. |
| 4. | Qi Han et al., China, 2021 | Cohort study | 43.3% | To test the hypothesis that neural oscillations obtained before surgery can predict acute postoperative pain seeing sustainable interaction between neural oscillations and pain perception. | Patients took part in the experiment with the following inclusion criteria: American Society of Anesthesiologists grade I-II, secondary school education, and age between 35 and 65 years old, and without: preoperative opioid users or chronic pain sufferers, postcraniotomy or traumatic brain injury, psychiatric family history or psychiatric diseases and neurological diseases | Acute Postoperative Pain | Between November 2018 and March 2019, patients admitted to Beijing University People's Hospital for wedge resection, lobectomy or mediastinotomy during thoracoscopy were recruited. | Neuronal oscillations can be used to predict acute postoperative pain before surgery and, in the future, this method may lead to better control of postoperative pain. |
| 5. | Atsushi Kimura et al., Japan, 2021 | Cohort study | 21.7% | To develop a system that uses machine learning to properly assess the level of pain throughout walking subjects by recording their quantitative electroencephalography (qEEG). | 23 patients who had total hip replacements for pain were included in the study: 5 males and 18 females, ranging in age from 44 to 80. | Hip pain | The sample size was small, and the authors only included patients with hip pain. Due to reports that THA patients have better postoperative outcomes than those who have total knee arthroplasty 45–47, the authors limited the study to THA patients. Furthermore, different factors, such as radiographic findings, such as a KL classification of 3 and restricted ADL of patients not caused by pain, serve as indications for THA surgery. | The developed system is a helpful tool for non-invasive and objective monitoring of patients' pain while performing ADL. |
| 6. | Jiahao Wang et al., China, 2020 | Case-control study | 69% | This study aimed to develop a machine learning (ML) model to predict the level of pain using extracted features. In this regard, the autoencoder (AE) model based on EEGNet was created to extract a set of features from pain-induced high-density EEG potentials. | This study included 29 participants with 20 males and 9 females, ranging in age from 17 to 25. All participants were informed of the written consent. Furthermore, there was no history of chronic pain among the participants. | Subjective Pain  Perception | No | The proposed method based on AE is more effective at identifying pain-related features and achieving better classification performance compared to traditional methods. |
| 7. | Gomutbutra, et al., Thailand, 2021 | Experiment | 55% | To estimate the accuracy and practicality of the model based on the data from OpenFace© to categorize pain intensity in elderly Asian patients. | 255 Thai individuals | Chronic pain | No | The accuracy of OpenFace© compared to human FACS coding is 90% for constrained images and 80% for everyday life images. However, the validity of OpenFace© in detecting a face in elderly individuals and dementia is troublesome. |
| 8. | Semwal and Londhe, India, 2021 | Experiment | no data | To explore automated pain detection using head position using CNN-BiLSTM network. | 16 subjects (26 000 samples) | Pain | No | The model showed high correlation between head pose and pain. The proposed system demonstrates high. effectiveness in pain prediction. |
| 9. | Kornprom Pikulkaew and Varin Chouvatut, Thailand, 2021 | Experiment | 48.84% | To suggest utilizing deep learning techniques for identifying and categorizing facial pain. | 129 subjects | Pain | No | The suggested method increased the accuracy and durability of pain detection utilizing neural networks, not just in monitoring cases but also in more complex environments. |
| 10. | S Jerritta et al., India, 2022 | Experiment | No data | To develop an algorithm for automatic detection of pain by analyzing ECG and EMG signals in the time-frequency domain using Daubechies wavelet, and also various features are extracted in the domain and classified using machine learning algorithms. | 90 subjects | Pain | No | HRV signals and EMG signals combined produce better results compared to HRV signals.  The 4th, 5th, and 7th orders of Daubechies wavelets can be considered to perform better than other Daubechies wavelets for a combination of HRV and EMG signals. |
| 11. | Kelati et al., Sweden, Tunisia, Saudi Arabia, 2022 | Experiment | No data | to classify pain level using a K-NN classifier and to propose a solution that can be implemented with IoT. | 85 subjects | Pain | No | Facial EMGs and individual emotional participation allowed to discriminate between the baseline and the pain tolerance level and to distinguish between pain and no pain. |
| 12. | Morabit et al., France, 2022 | Experiment | No data | To provide an effective pain assessment method based on facial expressions;  To present a fine-tuned data-efficient image transformers - deep learning networks - (Deit) for pain and no pain detection;  To classify pain into four classes: No pain, weak pain, mild pain and severe pain. | 25 adults (UNBC database)  87 subjects (BioVid Heat Pain Database) | Pain | No | Transformers: the size of the dataset improves results if there is a significant difference in size.  The GoogeNet-PNP achieved 80.01% accuracy while trained on the UNBC-McMaster shoulder pain [3] dataset.  The GoogleNet-PNP on the BioVid Heat Pain [4] dataset. In this case, the accuracy achieved was 65.75%. |
| 13. | Piri et al., Denmark, Sweden, 2022 | Case-control | 58.333% | To use artificial intelligence (AI)‐based quantification in order to compare PET scan results in two sex‐and age‐matched groups with low back pain and a similar control group of pain-free subjects. | 12 cases and controls | Low back pain | No | AI‐based quantification of relevant lumbar VOIs in PET/CT scans of LBP patients and controls is promising. |
| 14. | Soin et al., USA, 2022 | Prospective, observational pilot study | No data | To explore the possibility of using artificial intelligence via machine learning algorithms to analyze specific data points and to predict the most likely diagnosis related to spinal pain. | 246 patients | low back pain,  lumbar radiculopathy, post-laminectomy syndrome, and sacroiliitis | No | There is a promise for augmented decision-making with the help of artificial intelligence. |
| 15. | Kreiner & Viloria, Uruguay, 2022 | Case-control | No data | to test the diagnostic capabilities of a novel neural network (multilayer perceptron) in orofacial pain and TMD. | No info | Temporomandibular disorders (TMD) and orofacial pain | No | An artificial neural network can help dental clinicians diagnose several types of orofacial pain and dysfunction. |
| 16. | Gram et al., 2015, Denmark | Subanalysis of a clinical trial | 46.88% | to find out whether morphine responders and non-responders could be distinguished using a support vector machine (SVM) classification based on EEG data collected before the drug was administered.  Examine the model's robustness and repeatability using data from the same participants from a different recording day. | 32 patients | cold pressor test | No | Machine learning based on pre-treatment encephalographic activity has made it possible to separate morphine analgesia responders from morphine analgesia non-responders on an individual basis when traditional group statistical methods had failed. |
| 17. | Jerrita et al., 2022, India | Experimental study | No data | To analyze geometric features obtained from the mouth area using machine learning classifiers such as K Nearest Neighbor (KNN) Classifiers and Regression Trees for pain detection. | 5 random subjects | heat pain | No | Both classifiers generate an accuracy of 95%-98% in pain signal detection. |
| 18. | Ghosh et al., 2022, India | Experimental study | 48.8% | To use sentiment analysis system to detect pain sentiments by analyzing facial expressions. | 129 participants | shoulder pain | No | The proposed system is superior to competing methods in image-based sentiment analysis. Among LR, K Nearest Neighbor, decision tree, Random Forest, and SVM classifier, the proposed system has attained better performance for the SVM classifier. |
| 19. | Kristian et al., 2023, Indonesia | Experimental study | No data | To detect infant cries and pain levels using the machine-learning-based system. | 23 infants | pain | Videos were taken before and after surgery (mostly hernia surgery). | A voice feature is more applicable for pain detection than a facial expression. A combination of both can reach better outcomes. |
| 20. | Patania et al., 2022, Italy | Experimental | No data | To estimate the efficiency of the Graph Neural Network that uses the graph structure of fiducial points that can be observed on people’s faces. | 87 subjects | pain | No | The GNN method has a higher accuracy than the baseline models. |
| 21. | Prajod et al., Germany, 2022 | Experimental | No data | To examine the differences in the learned representations of models trained on  experimental pain (BioVid heat pain dataset) and clinical pain (UNBC shoulder pain dataset). | 25 participants + 87 participants | shoulder and heat pain | No | Explainable artificial intelligence can effectively distinguish between clinical and experimental pain detection models based on facial expressions. |
| 22. | Hu et al., USA, 2019 | Experiment | 38.1% | To create a foundation for technology that will enable the real-time visualization, measurement, and decoding of the cascade of distributed cortical processes that determine when and where clinical pain occurs. | 21 participants | Hypersensitive teeth | No | A neuroimaging-based AR/AI concept may make it possible to observe, exactly measure, and localize pain in the human brain in real time. |
| 23. | Xu et al., USA, 2018 | Experiment | 65.73% | 1. To train a machine learning model to identify pain using Action Units (AUs) encoded by the iMotions system.  2. To investigate the connection between iMotions-coded (automatically) and human-coded (manually) Aus. | 143 pediatric study participants | Post-surgical recovery | Laparoscopic appendectomy | Features recovered from iMotions AU codings are more sensitive to domain changes than features extracted from human-coded AUs, resulting in the creation of a straightforward technique that trains a projection from automated data onto a subspace of manual features. |
| 24. | Fontaine et al., France, 2022 | Prospective clinical diagnostic‑accuracy study | No data | To determine if a deep learning system could be useful in assessing the severity of pain in a real-world clinical setting, such as post-operative pain. | 1189 patients | - | Any kind of surgery | AI that analyzes facial expressions could help doctors identify and assess the severity of pain, especially in patients who are unable to adequately communicate their pain on their own. |
| 25. | Menchetti et al., USA, Turkey, 2019 | Experiment | No data | To create a useful computer vision and machine learning-based instrument that can accurately assess pain for clinical and research uses. | No data | - | No | Results demonstrate much better performance as compared to those attained using other established methods. It might prompt a medical professional or lay caregiver to administer pain management when thresholds set by the healthcare system are exceeded based on AUs for pain. |
| 26. | Lopez-Martinez et al., USA, Canada, 2019 | Experiment | 100% | To explore the application of functional near-infrared spectroscopy (fNIRS) for the identification of induced pain, employing optodes only placed on the frontal cerebral cortex for fNIRS signals. | 43 participants | - | No | Machine learning and fNIRS measurements of brain activity may be utilized to identify the existence of pain. It is possible to achieve high accuracy in distinguishing pain conditions by using only frontal channels. |
| 27. | Fabi et al., USA, 2022 | Experiment | No data | To analyze the racial bias using a machine learning model that has been taught to recognize when various action units associated with painful facial expressions are activated. | 50 face images | - | No | The findings showed that variations in the visual detectability of various facial muscle activations related to skin color or other racially dependent face characteristics exist. However, neither the greater exposure to own-race faces nor the perceptual detectability seems to be sufficient to account for the racial bias in pain perception. |
| 28. | Bargshady et al., Australia, 2020 | Experiment | No data | To propose a brand-new, improved deep neural network architecture for the efficient, four-level threshold identification of pain intensity from an image of a facial expression. | 25 subjects | Shoulder pain | No | The proposed classification algorithm was investigated as a potential tool for the detection of pain intensity in several classes from facial expression photos, and as a result, it can be used as an AI tool in medical diagnostics for automatic pain diagnosis and subsequent pain management of patients. |
| 29. | Bargshady et al., Australia, 2020 | Experiment | No data | To create an ensemble deep learning model (EDLM) that combines three-stream hybrid deep neural networks, using Convolutional Neural Networks (CNN) to extract facial image features, identify, and correctly classify the pain. | 20 subjects from the MIntPAIN database and 25 subjects from the UNBC-McMaster Shoulder Pain dataset | Shoulder pain | No | The suggested EDLM model may correctly categorize pain and produce multi-class pain levels for prospective applications in the field of medical informatics. |
| 30. | Cheema et al., 2025, the United States | Retrospective observational cohort study | No data | To identify prognostic variables associated with outcomes in chronic low back pain (CLBP) treatment using machine learning. | 2497 patients with CLBP. | CLBP | Non-recent-surgical CLBP | Bootstrap random forest can identify prognostic variables influencing outcomes in CLBP physical therapy. Predictive accuracy could help estimate prognosis and guide treatment planning. |
| 31. | Huang et al., 2025, China | Prospective cohort study | 136 males (39.88%) and 205 females (60.12%). | To develop and validate a multidimensional machine learning model to predict the risk of recurrence in patients who have recovered from chronic low back pain (CLBP). | 341 | Chronic low back pain (CLBP). | No surgical treatment was part of the patient cohort. | A multidimensional machine learning model (MLR-MDM) incorporating diverse clinical information, including MRI findings, showed robust predictive performance and outperformed the STarT Back Tool (SBT), which is a widely used prognostic tool. |
| 32. | He et al., 2025, China | Retrospective study | 140 were females and 125 were males | To develop and evaluate a deep learning convolutional neural network (CNN) model to detect lumbar disc herniation (LDH) on MRI images | 265 | Lumbar disc herniation (LDH) | Surgeries performed for lumbar disc herniation (LDH) | Deep learning convolutional neural networks (CNNs) effectively assist in diagnosing lumbar disc herniation (LDH) and help in surgical decision-making based on MRI images.  The AI-assisted diagnostic approach achieves higher accuracy (94.7%) compared to pure AI (93.4%) and pure human (91.7%) methods. Future work should include diverse datasets and integration of additional MRI views to improve model generalizability and diagnostic capabilities. |
| 33. | Mao et al., 2025, China | Retrospective study | 178 males and 840 females | To investigate whether artificial intelligence (AI) techniques could be used to automatically detect temporomandibular joint (TMJ) degenerative joint disease (DJD) on cone beam computed tomography (CBCT) images, and to achieve automated classification of DJD by identifying typical condylar radiographic signs such as erosion, osteophytes, sclerosis, and subchondral cysts with sufficient accuracy. | 1018 | Temporomandibular joint (TMJ) degenerative joint disease (DJD) | No | The deep learning model based on the YOLOv10 algorithm can accurately detect TMJ degenerative joint disease (DJD) on CBCT images.  The model can also differentiate typical radiographic signs of DJD, including erosion, osteophytes, sclerosis, and subchondral cysts, with acceptable accuracy. |
| 34. | Choi et al., 2025, Republic of Korea, Australia, USA | Retrospective study | No data | To develop and evaluate an artificial intelligence (AI) model for screening degenerative joint disease (DJD) of the temporomandibular joint (TMJ) by integrating TMJ panoramic radiography and joint noise data. | 3908 | Degenerative joint disease (DJD) | No | AI models integrating temporomandibular joint (TMJ) panoramic radiography with joint noise data (both clinician-detected crepitus and patient-reported noise) demonstrated promising performance in diagnosing degenerative joint disease (DJD), outperforming orofacial pain specialists in accuracy and consistency. The AI model that combined all joint noise data with imaging achieved the highest diagnostic performance, with an F1-score of 0.72. Further research with larger, multi-center datasets and evolving diagnostic criteria is needed. |
| 35. | De Araujo et al., 2025, Brazil | Diagnostic AI evaluation | No data | To test an AI-based virtual assistant (PAINe) using natural language processing (NLP) to differentiate odontogenic pain from temporomandibular dysfunction (TMD) pain. | 50 random scenarios were used against the validated questionnaire | Odontogenic and TMD pain | Not applicable | The PAINe AI chatbot showed 86% accuracy with a validated pain screening questionnaire. This suggests AI-assisted virtual assistants may support the differential diagnosis of preliminary dental pain efficiently. |
| 36. | Liawrungrueang et al., 2024, Thailand | Evaluation study | No data | To develop and evaluate a deep learning model using a convolutional neural network (CNN) with a YOLO (You Only Look Once) architecture to automatically detect and grade lumbar intervertebral disc degeneration (IDD) from sagittal T2-weighted MRI images based on the Pfirrmann grading system. | 2000 MRI scans of lumbar spines | Lumbar intervertebral disc degeneration (IDD) | No data | The study demonstrates promising results for AI-assisted classification of lumbar IDD, suggesting it could significantly transform diagnostic radiology and patient care, but calls for further research to enhance generalizability and clinical integration. |
| 37. | Sada et al.,2024, Kosovo, Australia | Prospective study | Only infant boys | To examine parents’ use of two pain assessment instruments—PainChek Infant (an AI-enabled tool) and the Observer-Administered Visual Analog Scale (ObsVAS)—to assess postoperative pain in infant boys after circumcision;  To evaluate the construct validity and clinical utility of these tools in the home setting. | 109 infant boys (69 parents) | Circumcision without any complications | Circumcision surgery | The PainChek Infant (AI-enabled) and ObsVAS (observer-based) pain assessment instruments demonstrated construct validity and clinical utility when used by parents to assess postsurgical pain in infants at home. |
| 38. | Ah-Yan et al.,2024, Canada | Qualitative comparative observational study | No data | To evaluate the ability of ChatGPT and Copilot, to provide medical advice about low back pain (LBP) to patients;  To assess the impact of including clinical context information on the quality of the answers generated by these models. | 21 evaluators who provided answers (18 physiatrists across Quebec and 3 physiatry residents) | Low back pain (LBP) | No data | ChatGPT and Copilot can provide reliable medical advice on LBP, irrespective of whether a detailed clinical context is provided or not, supporting their beneficial role in helping patient self-management of LBP.  Responses from AI are valid, but sometimes not specific enough for particular clinical cases. |
| 39. | C Areias et al., 2024, USA, Portugal | Prognostic study | Females - 56.24% (3445 out of 6125) males-, 43.54% (2667 out of 6125);  nonbinary (0.15%) or preferring not to answer (0.07%). | To develop an artificial intelligence (AI) tool to assist physical therapists (PTs) in predicting an individual’s potential to achieve clinically significant pain relief by the end of the digital care program;  To identify the predictors of pain nonresponse. | 6125 | Low back pain (LBP) | No data | The AI predictive tool can effectively identify patients less likely to achieve significant pain relief early during treatment. |
| 40. | Cascella et al.,2024, Italy, India | Feasibility study | No data | To develop an AI model capable of accurately detecting pain through facial expressions by combining the YOLOv8 object detection algorithm with facial Action Units (AUs) analysis. | 500 facial images | Pain | No data | The YOLOv8 object detection algorithm, combined with facial Action Units, can effectively detect pain expressions in real time, but future research is needed. |
| 41. | Muhaimil et al., 2024, India | Prospective case-control study. | 82 females, 118 males | To use machine learning (ML) and deep learning (DL) algorithms to predict low back pain (LBP) by analyzing T2-weighted MRI images of the lumbar spine. | 200 | Low back pain | No data | Both machine learning (ML) and deep learning (DL) models can effectively predict low back pain from T2-weighted MRI images of the lumbar spine. |
| 42. | Granviken et al., 2024, Norway | Cluster randomized controlled trial (RCT) | No data | To evaluate the effectiveness of a case-based reasoning (CBR) driven clinical decision support system (CDSS) in physiotherapy practice for musculoskeletal (MSK) pain. | 711 | Neck, shoulder, back, hip, knee, or complex pain | No data | The implementation of an AI-based CDSS in physiotherapy practice did not influence treatment practice or improve patient outcomes in this study. |
| 43. | Snene et al., 2024, Switzerland | Prospective pilot study | 100% males | To evaluate the feasibility of real-time assessment of pain among patients with dementia using physiological data collected from wearable devices combined with machine learning methods.  Develop a labeled dataset annotated with analogue observations for pain intensity assessed by usual scales and their matching physiological signals. | 20 (18 completed trial period) | Dementia, including Alzheimer's disease, vascular dementia, or mixed dementia. | No data | The study demonstrated that wearable technology combined with machine learning can estimate pain intensity and frequency in patients with dementia. |
| 44. | Zapata-Caballero et al., 2025, Mexico | Evaluation study | No data | To evaluate the responses provided by ChatGPT, to commonly encountered patient questions about chronic pelvic pain (CPP). | 9 queries were answered by ChatGPT. | Chronic pelvic pain (CPP) | Pelvic floor surgery | ChatGPT effectively provides accurate, relevant, and clear answers to patient questions on CPP, but has challenges in terms of completeness and consistency. AI should be used as a supplemental tool for medical consultation. |
| 45. | Temel et al., 2024, Turkey | Observational evaluation study | No human sample | To investigate the accuracy and consistency of lumbar radicular pain (LRP) patterns demonstrated by the AI system ChatGPT-4o (radiculopathy pain patterns (L4, L5, and S1) on an anatomical model). | No human sample | Lumbar radicular pain (LRP) | No data | ChatGPT currently lacks the capacity to accurately and consistently represent LRP pain patterns.  AI cannot adequately replicate human pain experiences. |
| 46. | Shahid et al., 2024, Pakistan, Turkey | Cross-sectional study | No data | To determine if machine learning decision models (Random Forest and Gradient Boosted) and neural networks (multilayer perceptron and radial basis function-neural networks) could accurately predict joint pain using biochemical data and clinical attributes such as age, gender, uric acid, C-reactive protein, and other blood and liver function parameters. | 650 | Joint pain | No data | The study recommends the use of AI platforms, such as machine learning models, in hospitals integrated with computerized medical systems for earlier and more accurate detection of joint pain. Early AI-based detection can aid in preventing more serious orthopedic complications.  Random Forest model showed the best overall prediction accuracy (97%) with minimal errors.  The MLP neural network achieved the highest accuracy (98%) among neural networks.  Uric acid is the top biochemical predictor for joint pain. |
| 47. | Giordano et al., 2024, Austria, Germany | Comparative secondary analysis | 78.5% female infants (11 out of 14 infants);  21.5% male infants (3 out of 14) | To evaluate neonatal pain responses during routine capillary blood sampling procedures, using both AI metrics (FaceReader 9) (such as arousal and valence) and pain ratings given by healthcare professionals. | 14 infants | Procedural pain during routine capillary blood sampling | No data | The study highlights the potential of AI technology to accurately detect and analyze neonatal facial expressions related to pain, correlating well with expert clinical assessments. AI can serve as an objective tool to improve pain recognition and assessment precision in newborns. |
